# Comprehensive immunoinformatics and bioinformatics strategies for designing a multi-epitope based vaccine targeting structural proteins of Nipah virus

**DOI:** 10.3389/fimmu.2025.1535322

**Published:** 2025-05-13

**Authors:** Shivangi Sharma, Pragya D. Yadav, Sarah Cherian

**Affiliations:** Indian Council of Medical Research (ICMR)-National Institute of Virology, Pune, Maharashtra, India

**Keywords:** Nipah virus (NiV), immunoinformatics, multi-epitope vaccine, molecular docking, molecular dynamics simulation, immune simulation

## Abstract

**Background:**

Nipah virus (NiV) is characterized by recurring outbreaks and causes severe neurological impact, leading to increased mortality rates. Despite the severity of the disease, there is no proven post-exposure treatment available, emphasizing the critical need for the development of an effective vaccine.

**Objective:**

This study was aimed at designing a multi-epitope based vaccine candidate based on an in-silico approach.

**Methods:**

NiV’s Structural proteins were screened for B and T-cell epitopes, assessing characteristics like antigenicity, immunogenicity, allergenicity, and toxicity. Two vaccine constructs (NiV_1 & 2) were designed using different adjuvants (Cholera toxin and Beta-defensin 3) and linkers and their predicted 3D structures were evaluated for interaction with Toll-Like Receptor TLR-3 using docking and molecular dynamics (MD) simulation studies. Finally, The potential expression of the vaccine construct in Escherichia coli (E. coli.) was verified by cloning it into the PET28a (+) vector and immune simulations were undertaken.

**Results:**

The study identified 30 conserved, antigenic, immunogenic, non-allergenic, and non-toxic epitopes with a broad population coverage. Based on the stability of vaccine construct in MD simulations results, NiV_1 was considered for further analysis. *In-silico* immune simulations of NiV_1 indicated a substantial immunogenic response. Moreover, codon optimization and in-silico cloning validated the expressions of designed vaccine construct NiV_1 in E. coli.

**Conclusion:**

The findings indicate that the NiV_1 vaccine construct has the potential to elicit both cellular and humoral immune responses. Additional *in vitro* and *in vivo* investigations are required to validate the computational observations.

## 1 Introduction

Nipah virus (NiV) is a highly pathogenic RNA virus with a negative sense genome, classified within the Paramyxoviridae family. The fruit bat is the primary natural carrier of this virus, and its transmission to other animals like pigs and humans, facilitates the spread of this zoonotic virus to a wider range of hosts (1, 2). It was initially identified as the causative agent for a severe encephalitis outbreak in Malaysia and Singapore in 1998-1999, and the case-fatality rate of exhibited was 40% (3). Since then, numerous consecutive outbreaks have been documented in multiple countries such as India, Bangladesh and Philippines, particularly within the South and Southeast Asia region (3–6). It was determined that the illness observed in Malaysia and Bangladesh was attributed to two distinct strains of the NiV, referred to as NiV M and NiV B, respectively. Further investigations demonstrated that the NiV B strain which has been responsible for more recent outbreaks in Bangladesh and India is more pathogenic (7). In a recent outbreak in September 2023, Kozhikode district in Kerala reported six confirmed cases, including two deaths (https://www.who.int/emergencies/disease-outbreak-news/item/2023-DON490) Compared to other viral outbreaks, NiV infection has resulted in a lower incidence of cases and reduced human transmission. To date, over 700 confirmed cases of NiV infection have been reported with a great mortality rate of around 50 to 75% (8), indicating the potential for NiV to contribute to public health emergencies (9). Consequently, it has been designated as one of the priority pathogens in the World Health Organization (WHO) R&D Blueprint list (10).

NiV infection is usually identified by symptoms such as muscle pain, flu-like manifestations including fever, cough, nausea, dizziness and headaches. In more advanced cases, it can lead to severe conditions such as acute encephalitis, systemic vasculitis, and respiratory complications (11–14). With a broad host range, high virulence, and significant morbidity and mortality, the NiV is classified as a Biosafety Level 4 virus (9).

NiV has an enveloped, single-stranded RNA genome with a negative sense that is non-segmented and approximately 18kb in length. Its genetic composition includes six structural proteins: nucleoprotein (N), phosphoprotein (P), matrix protein (M), fusion protein (F), attachment glycoprotein (G), and the large protein or RNA polymerase protein (L). Additionally, it encodes three non-structural proteins called C, W, and V (15). The non-structural proteins act as inhibitors of interferon signaling to suppress the host’s innate immune response. The six crucial structural proteins within the NiV—namely glycoprotein (G), fusion (F), Matrix (M), polymerase (L), nucleocapsid (N), and phosphoprotein (P) on the other hand, are in general more capable of inducing an immune response in the host (16). The G and F proteins play critical roles in attaching to host cells and facilitating virion entry, with G aiding attachment and F guiding membrane fusion (17). The virus utilizes Class B2/B3 Ephrin as an entry receptor, predominantly found in respiratory tracts and the vascular system, resulting in conformational changes induced by the G protein that activates the F glycoprotein. Conversely, L, N, and P are involved in viral replication processes (18); L forms a complex responsible for transcribing viral mRNA as part of RNA-dependent RNA polymerase activity. N encapsulates transcribed RNA while also regulating transcription processes (19), whereas P binds to both polymerase and N serving as a processivity factor (20). The interactions between P and N proteins, as well as L protein, occur separately before the formation of the RdRp complex, and these interactions play regulatory roles in the process (21). Additionally, gene M encodes matrix protein crucial for virus budding and assembly.

Considering the recent Covid-19 pandemic, it is clear that numerous countries lack the capacity to effectively manage abrupt viral outbreaks, underscoring the importance of increased attention and ongoing research to tackle potential future viral crises. As a result, research efforts are being directed toward the development of effective therapeutics and vaccines to combat this emerging pathogen. Diverse strategies have been employed in the development of a potent vaccine against NiV, including the use of viral vectors like vesicular stomatitis virus (22) and rhabdovirus (23), recombinant vaccines such as the recombinant measles virus vaccine expressing the envelope glycoprotein of NiV (24), and Nipah virus-like particles composed of several NiV proteins (8, 25) in various animal models. However, all these vaccine candidates are currently undergoing preclinical trials. Additionally, Remdesivir (26) and ribavirin (27) have been employed in treating NiV infections, but their clinical efficacy remains uncertain. The antimalarial drug chloroquine has also shown potential in an *in-vitro* system but failed to show its effectiveness against NiV infection in *in-vivo* ferret models (28). A recent development involves the use of human monoclonal antibody therapy (m102.4) as an immunotherapeutic treatment against NiV infections, aiming to prevent both new and existing infections. In addition to m102.4, other cross-reactive anti-henipavirus mAbs has been isolated and characterized: h5B3.1. In a ferret animal model, a humanized cross-reactive fusion-specific antibody (h5B3.1) has shown promise in blocking the conformational change of membrane fusion protein and effectively neutralizing NiV and HeV diseases (29). This finding adds credence to the possibility that mAb-based therapeutics could be utilized for prophylaxis or post-exposure therapy in individuals who have been exposed to NiV or HeV. Nonetheless, due to the need for cold chain storage and intravenous administration, it may not be the most feasible option for addressing outbreaks in field settings (30–32).The absence of approved vaccines or therapeutics for human use against the Nipah virus poses a significant challenge in effectively managing and controlling its spread.

Over the past few decades, there has been a significant revolution in the realms of bioinformatics and structural biology. Continuous updates in computational tools for genomic data analysis have played a pivotal role in fostering the development of novel approaches for potential vaccine design. Recent research has highlighted the development of multi-epitope vaccines using computational techniques in immunoinformatics, eliminating the need for pathogen cultivation, speeding up vaccine production (33). By utilizing bioinformatics tools and algorithms, researchers can analyze the proteomic data of the pathogen to identify potential epitopes that can elicit an immune response (34). These epitopes can then be combined to create a multi-epitope subunit vaccine construct, which has several advantages such as reduced risk of disease reemergence, enhanced immunogenicity, and improved stability compared to traditional whole-virus vaccines (35). This new approach has shown potential as a strong candidate for clinical trials and could prove effective in combatting viral infections (36). This study aims to design a multi-epitope subunit vaccine construct for NiV and evaluate it using in-silico and immunoinformatics approaches.

## 2 Material and methods

**Figure 1:**
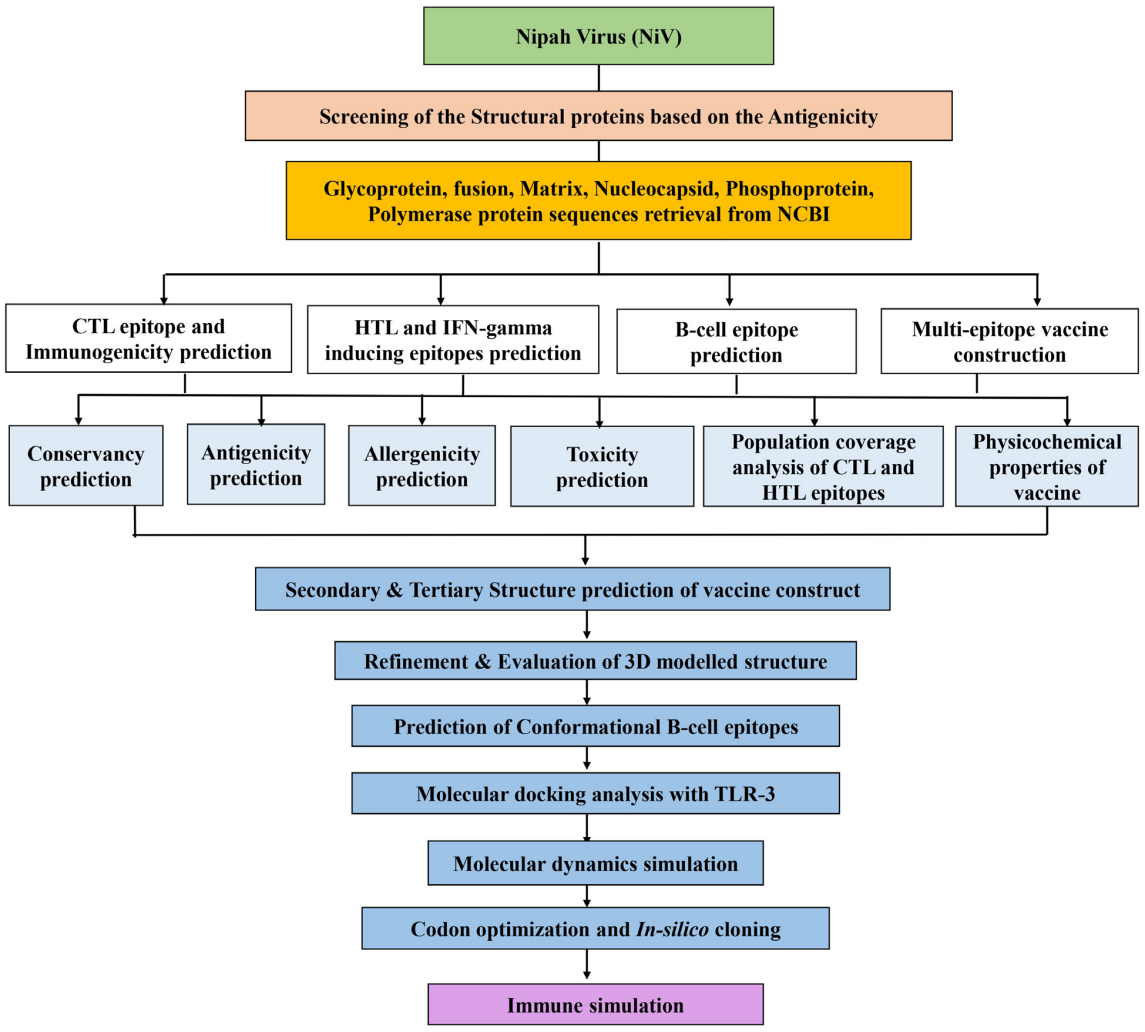
The workflow of the immunoinformatics guided design of the multi-epitope vaccine construct against NiV.

The sequential procedure followed in this work to develop and evaluate the multi-epitope vaccine candidate against NiV is shown in **Figure 1**.

### 2.1 Protein sequence retrieval and antigenicity prediction

The primary sequences of the G, F, M, L, P, and N proteins of NiV were obtained from the NCBI database (accessed on October 30, 2023) using the following accession IDs: NP_112027.1, NP_112026.1, NP_112025.1, NP_112028.1, NP_112022.1, and NP_112021.1. Their antigenicity was assessed using VaxiJen v.2.0 (accessed on October 30, 2023) (37). A threshold score of 0.4 was established as the cutoff for viral proteins, where proteins scoring higher than 0.4 were classified as antigenic, while those scoring less than 0.4 were deemed non-antigenic. Apart from the above protein sequences, the Cholera toxin subunit B and Beta-defensin 3 adjuvant protein sequences were obtained from the UniProt database, with the respective UniProt entry identifiers P01556 and Q5U7J2.

### 2.2 Prediction and screening of T-cell epitopes

The IEDB server (https://www.iedb.org/) was utilized for the identification of MHC-I/CTL and MHC-II/HTL epitopes within the six structural proteins of NiV (G, F, M, N, L, and P) (38). Cytotoxic T lymphocyte (CTL) and helper T lymphocyte (HTL) epitope predictions were conducted utilizing the NetMHCpan 4.0 EL and NetMHCIIpan 4.1 EL methods, respectively (39). For CTL epitopes, a peptide length ranging from 9 to 10 was taken into consideration, whereas for HTL epitopes, a length of 15 was utilized, both in conjunction with the reference set of alleles. The reference set of HLA alleles, signifying commonly shared binding specificities, was implemented to ensure population coverage exceeding 97% and 99% for MHC class I and II, respectively. Additionally, a rigorous criterion was established, with a percentile rank of less than 1 for MHC class I and less than 10 for MHC class II alleles, denoting robust binding peptides during the epitope screening. To validate the selected epitopes for their antigenicity, VaxiJen v.2.0 was employed, employing the default prediction value of 0.4 (37).

Moreover, the MHC I Immunogenicity Tool accessible at IEDB (http://tools.iedb.org/immunogenicity/) was utilized to project the immunogenicity of CTL epitopes (40). For further scrutiny, only CTL epitopes with positive immunogenicity score were taken into consideration. Conversely, HTL epitopes were analyzed for their potential to elicit IFN-γ cytokine production, contributing to the initiation of innate and adaptive immunity against the virus through the employment of the IFNepitope server (IFNepitope: A server for predicting and designing IFN-gamma inducing epitopes (osdd.net) (41). Epitopes demonstrating a “positive” response in terms of IFNγ release were exclusively selected for subsequent analysis.

### 2.3 B cell epitope prediction

To identify potential B cell epitopes within the NiV proteins, we utilized the BCPREDS server 1.1 (http://ailab-projects1.ist.psu.edu:8080/bcpred/predict.html), a web-based B cell epitope prediction tool (42). Epitope prediction focused on 16-mer peptides, with a selection threshold of prediction scores exceeding 0.90. The predicted epitopes were further subjected to antigenicity evaluation using the VaxiJen v2.0 server (http://www.ddg-pharmfac.net/vaxijen/VaxiJen/VaxiJen.html) (37).

### 2.4 Assessment of conservation, allergenicity, and toxicity of B & T-cell specific epitopes

The identified B-cell, CTL, and HTL epitopes underwent further analysis to refine the selection based on their conservation, allergenicity, and toxicity. To assess conservation levels, the IEDB (Epitope Conservancy Analysis at iedb.org) was employed for the conservation analysis of B and T-cell epitopes against the available sequences of both NiV strain B and M for each structural protein of NiV (G, F, M, N, L) (43). Only epitopes demonstrating 100% conservation were considered for subsequent property analysis.

AllerTop 2.0, accessible at http://www.pharmfac.net/allertop, was utilized to evaluate the allergenicity of B and T-cell epitopes. AllerTOP v2.0 utilizes auto- and cross-covariance (ACC) transformation, amino acid E-descriptors, and k nearest neighbor machine learning techniques for protein allergen classification. Epitopes identified as “probable non-allergenic” were specifically chosen for our analysis (44). Additionally, the ToxinPred online server, available at https://webs.iiitd.edu.in/raghava/toxinpred/index.html, was employed to predict the toxicity of B and T-cell epitopes (45).

### 2.5 Population coverage

The distribution of particular HLA alleles among various ethnicities and populations is crucial for developing an epitope-based vaccine. The IEDB population coverage tool (http://tools.iedb.org/population/) was utilized to assess the population coverage of the selected CTL and HTL epitopes across multiple HLA alleles in diverse global regions as well as on a worldwide level, calculating combined class I and II coverage (46).

### 2.6 Construction of multi-epitope based vaccine

The proposed design of the vaccine construct involved connecting an adjuvant to specific T and B-cell epitopes, which are interlinked by specific linkers to accurately define the epitopes. Various linkers utilized in this study, such as CTL linker (AAY), B epitope linker (KK), and HTL linker (GPGPG), are chosen to achieve solubility, improved expression levels, and precise folding of the multi-epitope structure (47, 48). Each multi-epitope vaccine was integrated with a potent immunostimulatory adjuvant to boost immunogenicity and activate both innate and adaptive immune responses (49). Two different adjuvants were employed in this study: CTB (Accession ID: P01556) and beta defensin (Accession ID: Q5U7J2). The adjuvant was linked to the N-terminal of the vaccine construct using the EAAAK linker (50). The final structure of the vaccine constructs consist of the adjuvant, along with CTL, B-cell, and HTL epitopes, arranged in that specified sequence. Following the assembly of the vaccine constructs, antigenicity was evaluated using VaxiJen v 2.0 (37), while their allergenicity was assessed using AllerTop 2.0 (44). Ultimately, the toxicity of the formulated vaccines were predicted using the ToxinPred web server (45).

### 2.7 Physiochemical properties prediction of vaccine construct

ExPASy’s ProtParam tool (https://web.expasy.org/protparam/) was used to analyze the physical and chemical characteristics of vaccine construct (51). A number of parameters were considered, including aliphatic index, stability index, Grand Average of Hydropathicity (GRAVY), and half-life. The instability index was calculated to determine the stability of epitopes *in vivo*, with a threshold of <40 indicating a stable vaccine (52). Additionally, SoluProt 1.0 (https://loschmidt.chemi.muni.cz/soluprot/) was used to evaluate the solubility of the vaccine (53).

### 2.8 2D and 3D structure prediction

The PSIPRED software (http://bioinf.cs.ucl.ac.uk/psipred/) was employed to predict the structures of alpha helix, beta helix, and coil in the vaccine constructs (54). The tertiary structure of the multi-target, multi-epitope vaccine peptide was generated using Robetta server (55), utilizing the RoseTTAFold algorithm for *de novo* protein modeling (https://robetta.bakerlab.org). RoseTTAfold is a tool that employs neural networks and simultaneously takes into account the arrangement of amino acids, their interactions, and the potential tertiary structures they can form (56).

### 2.9 Refinement and validation of 3D modeled structure of vaccine

To enhance the quality of the 3D modeled structure, a refinement process was applied using the GalaxyRefine online server (http://galaxy.seoklab.org/refine) (57). This process involves rebuilding and repacking of the side chains, followed by structure relaxation through molecular dynamics (MD) simulation. Validating the 3D structure after refinement provides an overview of the structure improvement and its current quality. Tools such as PROCHECK, the ERRAT tool from the UCLA-DOE LAB (https://saves.mbi.ucla.edu/) (58, 59), and the ProSA-web server (https://prosa.services.came.sbg.ac.at/prosa.php) were utilized to assess the validity and quality of the selected 3D structure (60).

### 2.10 Conformational B-cell epitope prediction

To identify potential epitopes capable of inducing B-cell production, the conformational B-cell epitopes were scrutinized for the final vaccine construct following 3D structure prediction and refinement. For the prediction of these epitopes, the ElliPro: Antibody Epitope Prediction tool from the IEDB database (tools.iedb.org/ellipro/) was utilized, leveraging protein structures or sequences to identify discontinuous antibody epitopes (61).

### 2.11 Molecular docking analysis

For analyzing the binding pattern of the multi-epitope vaccine polypeptide with receptors TLR-3, molecular docking analysis was carried out using the ClusPro 2.0 server (cluspro.bu.edu/login.php) (62). To facilitate this, TLR-3 (PDB ID: 1ziw) was obtained from RCSB PDB (RCSB PDB: Homepage) (63). ClusPro 2.0, an online server, employs a combination of shape-based and energy-based algorithms to predict the binding affinity of a protein-ligand complex. It analyzes the 3D structure of proteins, predicts binding sites, and identifies potential interactions between the proteins and ligands. Furthermore, to predict interacting residues involved in the molecular interactions of the designed vaccine construct and receptors, the online database PDBsum was utilized (64).

### 2.12 Molecular dynamics simulation

Examining the binding interactions between the vaccine and the receptor TLR3 requires considering their dynamic behavior. Consequently, MD simulations were conducted using the Desmond module from the Schrodinger suite (65, 66). The vaccine-TLR3 complex underwent simulation in a TIP3P predefined water solvent model within orthorhombic periodic boundary conditions. To achieve electrical neutralization, the addition of appropriate amounts of sodium and chlorine ions was performed. The system was then subjected to minimization under the OPLS4 force field (67). The NPT ensemble (68) was employed for the simulation, maintaining a constant temperature of 300 K at 1 atm pressure over a duration of 100 ns (69).

### 2.13 Codon optimization and in-silico cloning of vaccine construct

VectorBuilder Codon optimization tool, available at https://en.vectorbuilder.com/tool/codon-optimization, was utilized to evaluate the expression level of the multi-epitope vaccine in E. coli (strain K12). VectorBuilder conducted analyses for the GC content and Codon Adaptation Index value of the query sequence with the goal of achieving optimal expression. Subsequently, the final vaccine constructs were inserted into the pET-28a(+) plasmid using SnapGene software v5.2.3 (https://www.snapgene.com/).

### 2.14 Immune response simulation

To assess the capability of the designed vaccine to induce a sustained immune response, C-IMMSIM analyses were conducted using the webserver accessible at https://kraken.iac.rm.cnr.it/CIMMSIM/index.php?page=1. The analysis included the calculation of an immunogenic response following a single-dose injection. C-ImmSim employs an agent-based model incorporating machine learning techniques and immune epitope prediction. It utilizes a position-specific scoring matrix (PSSM) to enable the prediction of immune interactions (70).

## 3 Results

### 3.1 Primary analysis of the candidate sequences

Antigenicity is a crucial parameter that plays a significant role in the evaluation of proteins for vaccine development. Based on antigenicity screening of the complete amino acid sequences of the six NiV structural proteins (Glycoprotein, Fusion, Matrix, Nucleocapsid, Phosphoprotein, and Polymerase) using VaxiJen v.2.0 (37), all structural proteins (G, F, M, N, P, L) were projected to be antigenic with scores exceeding the 0.4 threshold, as demonstrated in **Table 1**. Consequently, these proteins were chosen for B- and T-cell epitope prediction and vaccine development.

**Table 1:** List of structural proteins with accession IDs and their respective VexiJen scores as predicted by the VexiJen v.2.0 server.

NiV’s structural protein	Accession ID	VexiJen Score	Prediction
Glycoprotein(G)	NP_112027.1	0.5110	Probable antigen
Fusion Protein (F)	NP_112026.1	0.5012	Probable antigen
Matrix Protein (M)	NP_112025.1	0.4033	Probable antigen
Nucleocapsid Protein (N)	NP_112021.1	0.5713	Probable antigen
Phosphoprotein (P)	NP_112022.1	0.5866	Probable antigen
Polymerase Protein (L)	NP_112028.1	0.4757	Probable antigen

### 3.2 Prediction of CTL and HTL epitopes

The aim of T-cell epitope prediction is to identify the shortest peptide sequences within an antigen that can activate either CD4 or CD8 T-cells (71). Antigen-presenting cells display T-cell epitopes on their surface where they attach to MHC class I or MHC-II proteins. T-cell epitopes that bind to MHC class I proteins are acknowledged by CD8 T-cells which differentiate into cytotoxic T lymphocytes (CTL), whereas those presented by MHC class II are recognized by CD4 T-cells, which are identified as helper T-cells (72).

The IEDB server was employed to analyze the antigenic sequences of all structural proteins using the NetMHCpan 4.0 EL and NetMHCIIpan 4.1 EL methods for identifying T-cell epitopes (CTL and HTL), with the HLA reference set. The selection of the complete HLA reference set aimed to generate epitopes that cover a broad range of globally prevalent MHC class I and II alleles. Based on the selected threshold for CTL and HTL, NetMHCpan 4.0 EL predicted 778 epitopes for G protein, 756 for F, 453 for M, 713 for N, 665 for P, 4569 for L protein. Similarly, NetMHCIIpan 4.1 EL used for HTL epitope prediction, identified 1312 epitopes for G, 1273 for F, 840 for M, 1290 for N, 1650 for P, and 5831 for L. Subsequent to these predictions, the most appropriate CTL and HTL epitopes were selected based on criteria including antigenicity, non-toxicity, complete conservation across all strains, and absence of allergenic properties, while also taking into account the frequency of interactions with various alleles. The immunogenic potential and ability to elicit cytotoxic T-cell responses were evaluated, employing the MHC class I Immunogenicity tool available in the IEDB server for CTL-specific epitopes. The IFNepitope server was utilized to examine the capacity to stimulate IFN-gamma production for HTL-specific epitopes. In consideration of these parameters, the top two epitopes for each protein were selected. A total 12 CTL and 12 HTL epitopes were considered for the designing of the vaccine construct, as detailed in **Tables 2**, **3**.

**Table 2:** The shortlisted non-allergic, non-toxic, immunogenic and conserved CTL epitopes (with respective antigenic score) for designing multi-epitope vaccine construct.

Protein’s Name	Position	Predicted Epitope	Antigenicity Score (on Vexijen v2.0)	Alleles
G	456	ASFSWDTMIK	0.451	HLA-A*11:01,HLA-A*03:01,HLA-A*30:01
	530	QTAENPVFTV	0.452	HLA-A*68:02,HLA-A*02:06
F	126	AQITAGVALY	0.653	HLA-B*15:01,HLA-A*30:02,HLA-B*44:03,HLA-A*26:01
	310	SIVPNFILV	0.575	HLA-A*02:06,HLA-A*68:02,HLA-A*02:01,HLA-A*02:03,HLA-A*26:01
M	88	TIAAYPLGV	0.8	HLA-A*02:03,HLA-A*02:01,HLA-A*02:06,HLA-A*68:02
	277	HIKINGVISK	0.452	HLA-A*03:01,HLA-A*11:01,HLA-A*68:01,HLA-A*30:01
N	199	QQKRVNPFF	1.695	HLA-B*15:01,HLA-A*32:01,HLA-A*30:02,HLA-A*23:01,HLA-A*24:02,HLA-B*44:02
	327	YPLLWSFAM	0.867	HLA-B*35:01,HLA-B*53:01,HLA-B*07:02,HLA-B*51:01,HLA-B*08:01
P	398	KSRGIPIKK	1.397	HLA-A*30:01,HLA-A*03:01,HLA-A*31:01,HLA-A*11:01
	532	RLNHIEEQV	0.553	HLA-A*02:01,HLA-A*02:03,HLA-A*02:06,HLA-A*32:01
L	682	HTEFNPHNHY	1.096	HLA-A*01:01,HLA-A*30:02,HLA-A*26:01,HLA-B*44:03,HLA-B*44:02,HLA-B*15:01,HLA-B*57:01,HLA-A*68:01,HLA-B*58:01,HLA-B*40:01,HLA-A*32:01
	807	TIATIPFLF	0.932	HLA-A*23:01,HLA-A*24:02,HLA-A*26:01,HLA-A*30:02,HLA-A*32:01,HLA-B*58:01,HLA-B*57:01,HLA-B*53:01,HLA-B*35:01,HLA-B*15:01

G, Glycoprotein; F, Fusion; M, Matrix; N, Nucleocapsid; P, Phosphoprotein; L, Polymerase.

**Table 3:** The shortlisted non-toxic, non-allergic, IFN-γ inducing and conserved HTL epitopes (with respective antigenic score) for designing multi-epitope vaccine construct.

Protein’s Name	Position	Predicted Epitope	Antigenicity Score (on Vexijen v2.0)	Alleles
G	507	VYNDAFLIDRINWIS	0.5724	HLA-DRB3*01:01,HLA-DRB3*02:02,HLA-DQA1*01:01/DQB1*05:01,HLA-DPA1*01:03/DPB1*04:01,HLA-DRB1*04:01,HLA-DRB1*03:01,HLA-DPA1*03:01/DPB1*04:02
	515	DRINWISAGVFLDSN	0.7481	HLA-DRB1*04:05,HLA-DQA1*03:01/DQB1*03:02,HLA-DRB1*09:01,HLA-DRB1*12:01,HLA-DQA1*05:01/DQB1*03:01,HLA-DQA1*05:01/DQB1*02:01,HLA-DRB1*07:01,HLA-DRB1*04:01,HLA-DPA1*02:01/DPB1*01:01
F	512	FISFIIVEKKRNTYS	1.4463	HLA-DRB1*13:02,HLA-DRB1*11:01,HLA-DRB1*08:02,HLA-DPA1*02:01/DPB1*05:01,HLA-DRB1*03:01,HLA-DRB1*12:01,HLA-DPA1*02:01/DPB1*01:01,HLA-DRB5*01:01,HLA-DPA1*01:03/DPB1*02:01
	522	RNTYSRLEDRRVRPT	0.5681	HLA-DQA1*01:01/DQB1*05:01,HLA-DRB1*01:01,HLA-DQA1*04:01/DQB1*04:02,HLA-DRB1*04:05,HLA-DRB5*01:01,HLA-DRB1*04:01,HLA-DRB1*07:01,HLA-DRB1*15:01,HLA-DRB1*11:01,HLA-DRB1*09:01,HLA-DRB3*02:02
M	82	KRKKIRTIAAYPLGV	0.4099	HLA-DRB1*12:01,HLA-DPA1*02:01/DPB1*05:01,HLA-DRB1*15:01,HLA-DQA1*03:01/DQB1*03:02,HLA-DQA1*01:01/DQB1*05:01,HLA-DQA1*05:01/DQB1*02:01,HLA-DRB1*07:01,HLA-DRB1*01:01,HLA-DPA1*02:01/DPB1*01:01,HLA-DQA1*04:01/DQB1*04:02,HLA-DPA1*02:01/DPB1*14:01,HLA-DRB5*01:01,HLA-DRB1*09:01,HLA-DPA1*03:01/DPB1*04:02,HLA-DRB1*13:02,HLA-DPA1*01:03/DPB1*04:01,HLA-DQA1*05:01/DQB1*03:01,HLA-DRB3*02:02,HLA-DPA1*01:03/DPB1*02:01,HLA-DRB3*01:01,HLA-DRB4*01:01,HLA-DRB1*04:05,HLA-DRB1*08:02,HLA-DRB1*04:01
	183	DSGIYMIPRTMLEFR	0.6015	HLA-DRB1*12:01,HLA-DRB1*13:02,HLA-DRB1*11:01,HLA-DRB1*03:01,HLA-DRB1*08:02,HLA-DPA1*02:01/DPB1*05:01
N	23	ASFRSYQSKLGRDGR	0.4554	HLA-DQA1*04:01/DQB1*04:02,HLA-DRB1*15:01,HLA-DRB1*08:02,HLA-DRB5*01:01,HLA-DRB1*13:02,HLA-DRB1*11:01,HLA-DRB1*04:01,HLA-DRB1*04:05,HLA-DRB1*07:01,HLA-DRB1*09:01,HLA-DRB1*01:01,HLA-DQA1*01:01/DQB1*05:01
	187	WILIAKAVTAPDTAE	0.4879	HLA-DRB1*04:05,HLA-DQA1*03:01/DQB1*03:02,HLA-DRB1*09:01,HLA-DQA1*05:01/DQB1*03:01,HLA-DRB1*04:01,HLA-DQA1*01:02/DQB1*06:02,HLA-DRB1*01:01
P	248	LEFEDEFAGSSSEVI	0.7232	HLA-DRB1*09:01,HLA-DRB1*07:01,HLA-DQA1*05:01/DQB1*02:01,HLA-DQA1*05:01/DQB1*03:01,HLA-DQA1*03:01/DQB1*03:02,HLA-DRB3*01:01
	518	MGVINSIKLINLDMR	1.4813	HLA-DPA1*03:01/DPB1*04:02,HLA-DRB1*12:01,HLA-DPA1*02:01/DPB1*01:01,HLA-DQA1*05:01/DQB1*02:01,HLA-DPA1*01:03/DPB1*04:01,HLA-DPA1*01:03/DPB1*02:01,HLA-DRB4*01:01
L	253	KSDIKYQPLISRSNA	0.8108	HLA-DPA1*02:01/DPB1*05:01,HLA-DRB4*01:01,HLA-DRB5*01:01,HLA-DPA1*03:01/DPB1*04:02,HLA-DQA1*04:01/DQB1*04:02,HLA-DPA1*02:01/DPB1*01:01,HLA-DRB1*12:01,HLA-DRB1*03:01,HLA-DRB1*01:01,HLA-DRB1*13:02,HLA-DPA1*01:03/DPB1*02:01,HLA-DPA1*01:03/DPB1*04:01,HLA-DPA1*02:01/DPB1*14:01,HLA-DRB1*04:05,HLA-DRB1*08:02,HLA-DRB3*02:02,HLA-DRB1*11:01,HLA-DRB1*04:01
	1972	LLVSKIAYTPGFPIS	0.518	HLA-DRB1*07:01,HLA-DRB1*09:01,HLA-DRB1*15:01,HLA-DPA1*02:01/DPB1*01:01,HLA-DRB1*12:01,HLA-DPA1*02:01/DPB1*14:01,HLA-DPA1*01:03/DPB1*04:01,HLA-DPA1*03:01/DPB1*04:02,HLA-DRB1*01:01,HLA-DPA1*02:01/DPB1*05:01,HLA-DRB1*13:02,HLA-DRB3*01:01,HLA-DRB5*01:01,HLA-DRB3*02:02,HLA-DPA1*01:03/DPB1*02:01

G, Glycoprotein; F, Fusion; M, Matrix; N, Nucleocapsid; P, Phosphoprotein; L, Polymerase.

### 3.3 Identification and analysis of linear B cell epitopes

The role of B-cell epitopes is vital in the multi-epitope vaccine as they trigger B-lymphocytes to generate antibodies, a crucial aspect of adaptive immunity. To predict linear B-cell epitopes from all structural proteins, the BCPREDS tool was utilized. Following prediction, the identified epitopes were rigorously assessed for antigenicity, non-allergenicity, and non-toxicity. Epitopes that met these criteria and exhibited high antigenicity scores were considered suitable. A total of six B-cell epitopes, as detailed in **Table 4**, were shortlisted from the six structural proteins for inclusion in the vaccine construct. Every chosen epitope from each protein exhibited complete identity with all retrieved amino acid sequences of the respective protein. Therefore, the shortlisted epitopes were predicted to possess a cross-reactivity against both NiV M and B strains.

**Table 4:** List of the shortlisted linear B-cell epitopes with properties for the vaccine construct design.

Protein’s name	Position	Predicted epitope	Score	Antigenicity	Allergenicity	Toxicity	Conservancy (%)
G	25	IKSYYGTMDIKKINEG	0.951	Probable antigen	Non-allergen	Non-toxin	100
F	214	FGPNLQDPVSNSMTIQ	0.972	Probable antigen	Non-allergen	Non-toxin	100
M	190	PRTMLEFRRNNAIAFN	0.956	Probable antigen	Non-allergen	Non-toxin	100
N	112	PVMERRGDKAQEEMEG	1.00	Probable antigen	Non-allergen	Non-toxin	100
P	238	YTSDDEEADQLEFEDE	0.996	Probable antigen	Non-allergen	Non-toxin	100
L	537	VSYSLKEKETKQAGRL	0.992	Probable antigen	Non-allergen	Non-toxin	100

G, Glycoprotein; F, Fusion; M, Matrix; N, Nucleocapsid; P, Phosphoprotein; L, Polymerase.

### 3.4 HLA population coverage analysis

The distribution of MHC HLA alleles varies across different geographic regions and ethnic populations worldwide. Thus, it is important to consider population coverage when designing a vaccine for maximum effectiveness. In this study, totally 24 (12 CTL and 12 HTL) identified epitopes were analyzed for population coverage at the worldwide level using IEDB population coverage tool. Epitopes identified to bind to multiple MHC alleles are deemed optimal only if their combined frequency within a population demonstrates substantial coverage, nearing 100% or achieving close to it. This is important for ensuring that a vaccine targeting those epitopes would be effective in a majority of individuals within the population.

**Figure 2:**
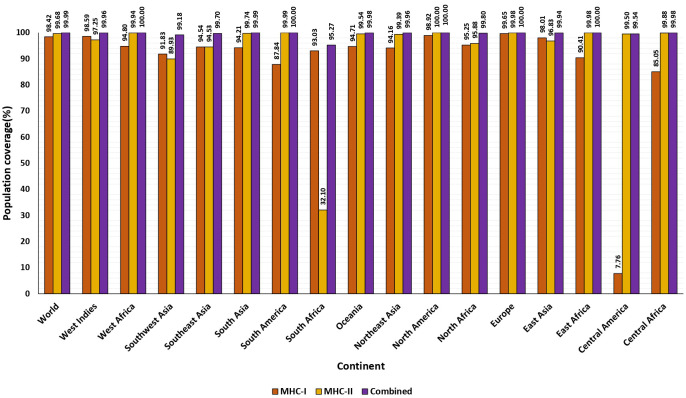
The percentage of population covered by HLA binding alleles associated with selected epitopes. The worldwide and average population coverage percentages for chosen epitopes of MHC class I and II. The illustration shows MHC class I in orange, MHC class II in yellow, and the combined MHC class I and MHC class II in purple.

The shortlisted CTL and HTL epitopes covered 98.42%and 99.68% of the global population, respectively. Overall, the significant population coverage was observed for the chosen 24 epitopes. These epitopes were found to cover 99.99% of resultant alleles of the world population (**Supplementary Figure 1**). The highest coverage of 100% was identified across five distinct regions worldwide, spanning East and West Africa, Europe, and North and South America, as determined by the combined CTL and HTL epitopes analysis. Additionally, during population coverage prediction, we noted the prevalence in regions highly impacted with previous Nipah outbreaks: South, Southeast Asia. The population data for these regions encompassed the global population, indicating potential extensive coverage for countries like India, Bangladesh, Malaysia, Singapore, and the Philippines, where Nipah outbreaks are common. South Asia indicated a coverage rate of 99.99%, with strong CTL and HTL coverage rates of 94.91% and 99.74% respectively. Conversely, Southeast Asia also demonstrated a strong coverage rate of 99.70% for combined CTL and HTL epitopes (**Figure 2**).

### 3.5 Design and construction of the multi-epitope vaccine candidate

**Figure 3:**
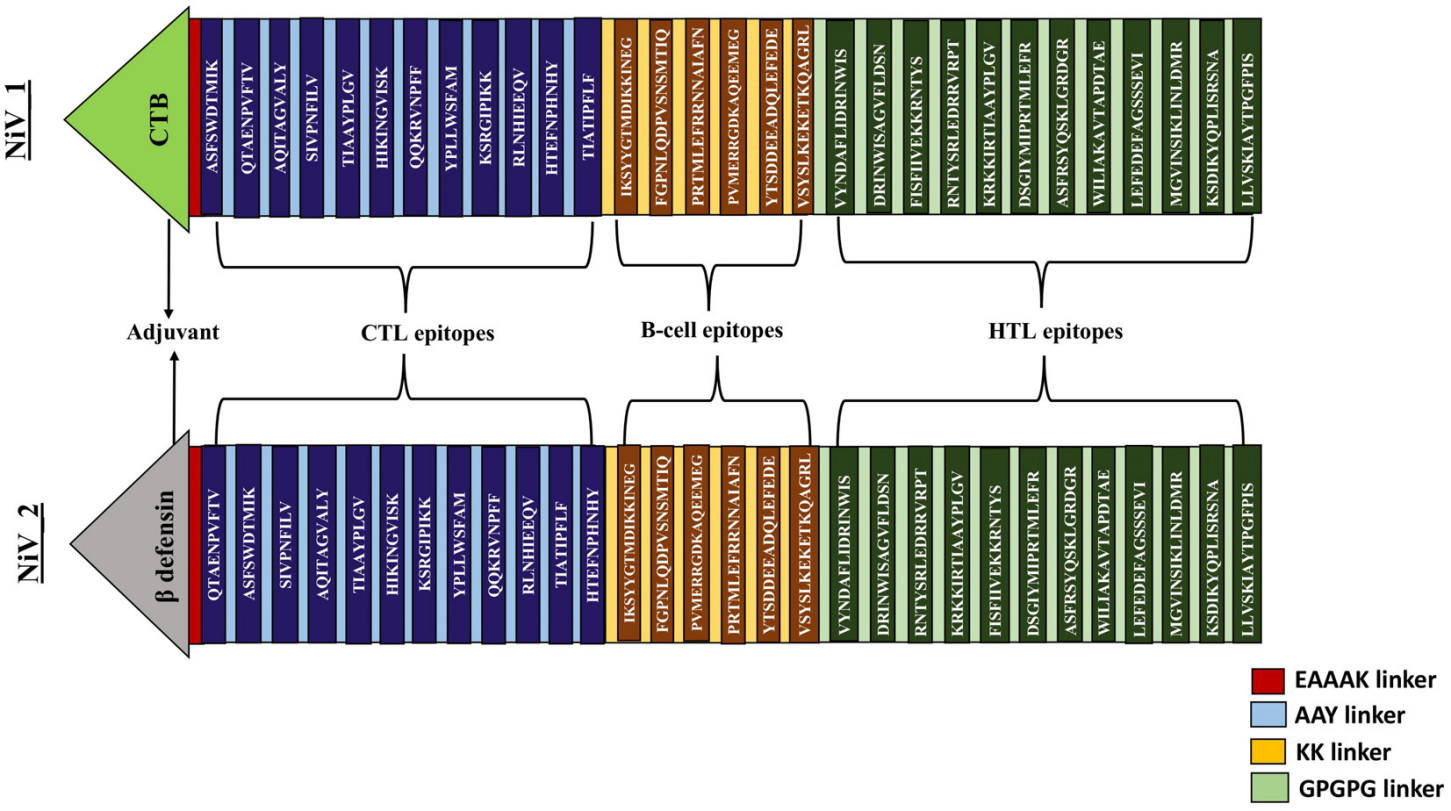
Systematic representation of proposed vaccine constructs (NiV_1 & 2). By utilizing an EAAAK linker, an adjuvant was introduced at the N-terminal tail of the vaccine, followed by the fusion of 12 CTL, 6 BCL, and 12 HTL epitopes connected by AAY, KK and GPGPG linkers.

Two distinct constructs were designed by integrating antigenic epitopes with two different adjuvants (Cholera Toxin B, and Beta-defensin) and linkers. While CTB and Beta-defensin 3 may pose potential risks to vaccine efficacy and immune response, these can be mitigated through optimized dosing and controlled delivery (73, 74). In this study, CTB was chosen over whole Cholera Toxin due to its non-toxic nature and its ability to enhance immune activation (75). Given the impracticality of evaluating numerous potential epitope arrangements, we selected representative constructs for the analysis. The sequences of these constructs differed according to the adjuvant employed and the arrangement of the epitopes. These constructs, comprising 12 CTL, 6 B-cell, and 12 HTL epitopes, were interconnected using universal linkers (EAAAK, AAY, KK, and GPGPG) as represented in **Figure 3** (47, 48).

### 3.6 Antigenicity, allergenicity and toxicity evaluation

An ideal vaccine should exhibit antigenicity, be non-allergenic, and free from toxins. Both vaccine candidates (NiV_1-2) were predicted to be antigenic by the VaxiJen v2.0 server. Furthermore, analysis through the AllerTOP v2.0 and ToxinPred servers indicated that the constructs are likely non-allergenic and non-toxic, respectively.

### 3.7 Physicochemical properties

The physicochemical properties were assessed using various parameters, including amino acid composition, molecular weight, theoretical pI, estimated half-life, instability index (I.I), aliphatic index (A.I), and the grand average of hydropathicity (GRAVY), as illustrated in **Table 5**. The lengths of the vaccine constructs NiV_1 and NiV_2 consist of 623 and 544 amino acids, respectively, with predicted molecular weights ranging from 59.37 to 68.35 kDa. Both constructs are classified as basic, with theoretical pI values between 9.50 and 9.69. Among the two constucts, NiV_2 had the highest predicted solubility (0.82) and theoretical pI (9.69), as well as elevated antigenicity. For both NiV_1 and NiV_2 constructs, the estimated half-life in mammalian reticulocytes, representing an *in vitro* environment, is approximately 30 hours, while in yeast it is predicted to exceed 20 hours and in Escherichia coli over 10 hours, simulating *in vivo* conditions. The instability index is calculated, indicating protein stability. The GRAVY score ranges from -0.321 to -0.407, reflecting the hydrophilic nature of the vaccine constructs, suggesting favorable interaction with surrounding water molecules. In summary, the additional physicochemical parameters of the proposed multi-epitope vaccine candidates fall within acceptable ranges, reinforcing their potential as viable vaccine candidates.

**Table 5:** The Physicochemical properties of the proposed multi-epitope vaccine.

Property	NiV_1	NiV_2	Indication
Antigenicity	0.59	0.6071	Antigenic
Allergenicity	Non-Allergen	Non-Allergen	Non-Allergen
No. of Amino Acids	623	544	Appropriate
Formula	C_3096_H_4817_N_829_O_883_S_17_	C2683H4199N739O761S17	Appropriate
Molecular weight	68325.35	59529.37	Appropriate
Total number of -ve charged residues (Asp + Glu)	56	47	–
Total number of +ve charged residues (Arg + Lys)	78	77	–
Theoretical pI	9.5	9.69	Basic
Estimated Half Life:			Appropriate
mammalian reticulocytes, *in-vitro*	30 hours	30 hours	
yeast, *in-vivo*	>20 hours	>20 hours	
Escherichia coli, *in-vivo*	>10 hours	>10 hours	
Instability Index	30.75	32.68	Stable
Aliphatic Index	76.68	72.72	Thermostable
Grand average of Hydropathicity (GRAVY)	-0.321	-0.407	Hydrophilic
Toxicity of Vaccine Construct	Non-toxic	Non-toxic	Non-toxic
Solubility	0.788	0.82	Soluble

### 3.8 2D and 3D structure prediction, refinement and evaluation

The stability of the vaccine candidates in real-world conditions was evaluated by using the 623 and 544 amino acid peptide sequences to predict both the secondary and tertiary structures. According to results from the PSIPRED server, the secondary structure of the designed vaccine constructs comprises numerous helices, a small number of strands, and a significant portion of coils (**Supplementary Figures 2A, B**). The secondary structure analysis of NiV_1 and NiV_2 revealed composition of 33.70% and 30.69% helices, 48.31% and 49.09% coils, and 17.97% and 20.22 strands, respectively.

**Figure 4:**
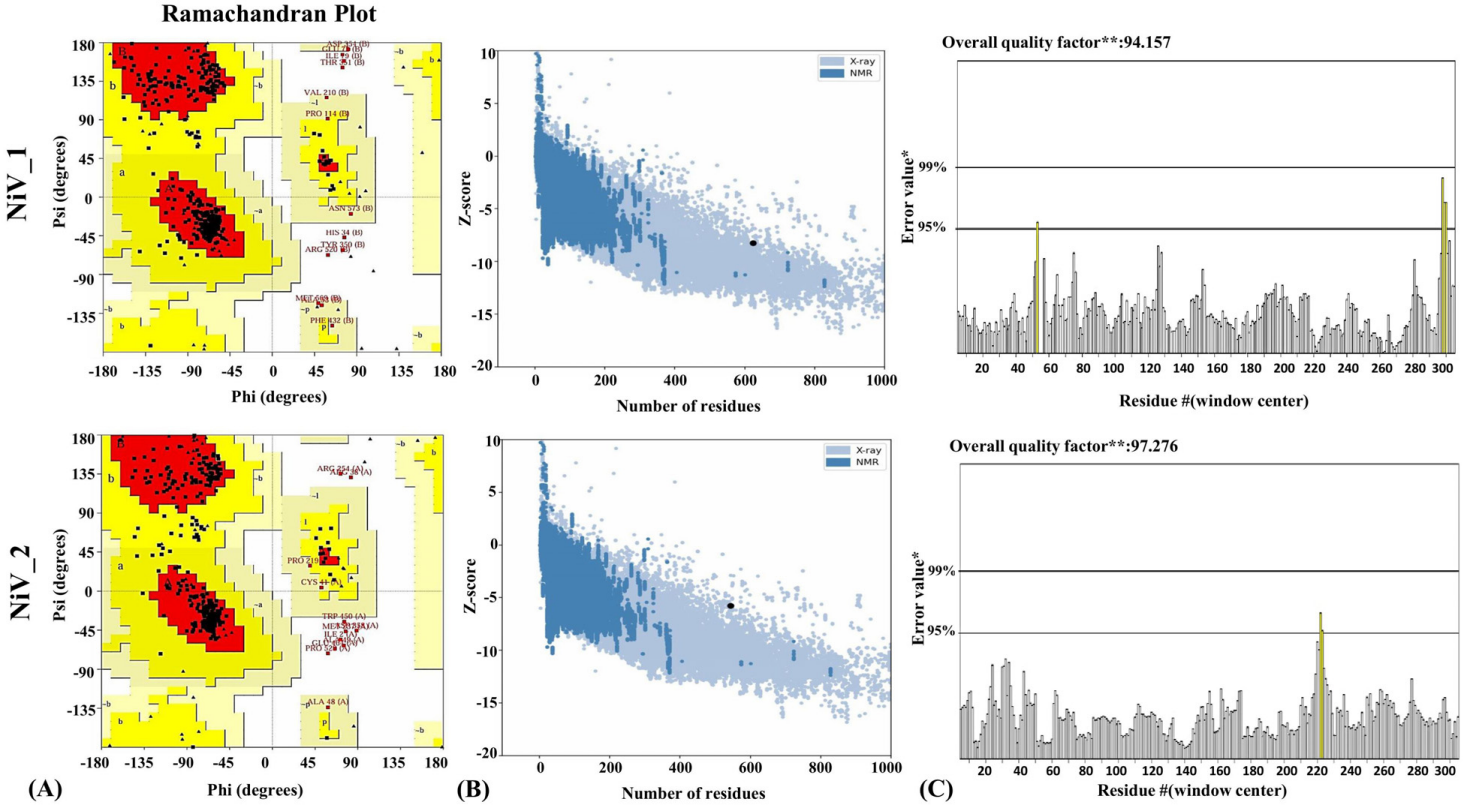
Structural analysis of refined 3D models of NiV_1 & 2 vaccine constructs. **(A)** Validation of refined 3D structure of vaccine construct by Ramachandran plot analysis **(B)** Validation of the refined model utilizing ProSA-web **(C)** Additionally, ERRAT was employed to evaluate the overall quality factor of the refined structure, indicating the high model quality.

The Robetta server, employing the RoseTTAFold algorithm, was utilized to predict the tertiary structure of the proposed vaccines, resulting in five potential 3D structures. The optimal model for the vaccines was selected based on criteria that included having the highest proportion of residues in the favorable regions of the Ramachandram plot and the lowest percentage in the outlier region, indicating superior structural quality. Model 1 and 3 of NiV_1 and NiV_2 emerged as the best among the RoseTTAFold-generated models. For NiV_1, the Ramachandran plots illustrating that 88.6% of residues lie in the most favored region, 9.1% in the allowed region, and only 1.2% in the disallowed region (**Supplementary Figure 3**). To further enhance the stability of the predicted structure and increase the number of residues in the favorable region, the structure was subjected to refinement using the GalaxyRefine server. The refined structure was validated using the Ramachandran plot via the PROCHECK server, revealing notable improvements. In the best model of NiV_1 (**Figure 4A**), 91.3% of residues fell within the favored region, with 6.4% in the allowed region, and only 1.4% in the outlier region.

Conversely, the model of the NiV_2 exhibited 87.7%, 9.8%, 0.9%, 1.6% residues in the most favored, additionally allowed, generously allowed, and disallowed regions, respectively (**Supplementary Figure 3**). The refined model of NiV_2 demonstrated 90.5%, 7.3%, 0.5%, 1.8% residues in the Ramachandran favored, additionally allowed, generously allowed, and disallowed regions, respectively (**Figure 4A**). Furthermore, ProSA-web analysis was conducted to assess quality and identify potential flaws in the top models. This analysis computes z-scores, which validate the modeled protein’s alignment with similarly sized natural proteins determined by NMR or X-ray methods. The initial models of the NiV_1 and NiV_2 yielded a z-score of -8.17 and -5.73, which changed to -8.27 and -5.78 post-refinement, respectively (**Figure 4B**). These z-scores fall within the acceptable range for protein structures of similar size. Moreover, the overall quality factor of the designed vaccine models, as determined by ERRAT analysis, were 94.155 and 97.276, indicating a high-quality model suitable for further analysis (**Figure 4C**), including molecular docking, and simulations. ProSA-web Z-score and Ramachandran plot details of NiV_1**–**2 are provided in **Figure 4** and **Table 6**.

**Table 6:** Comparative analysis of modeled tertiary structures of vaccine constructs: Pre- and Post-validation.

Vaccine	Ramachandran plot								Prosa		ERRAT score	
	Most favored regions		Additional allowed regions		Generously allowed regions		Disallowed region		z-Score			
	Before	After	Before	After	Before	After	Before	After	Before	After	Before	After
NiV_1	88.60%	91.30%	9.1%	6.4%	1.2%	1.0%	1.2	1.4	-8.17	-8.27	94.90%	94.15%
NiV_2	87.70%	90.50%	9.8%	7.3%	0.9%	0.5%	1.6	1.8	-5.73	-5.78	93.38%	97.27%

NiV_1 and NiV_2 represent vaccine construct 1 and vaccine construct 2, respectively.

### 3.9 Screening of conformational B cell epitopes

The 3D structures of the proposed vaccine candidates, NiV_1 and NiV_2, were screened for conformational B-cell epitopes using the ElliPro webserver at 0.5 threshold, identifying 7 and 9 epitopes, respectively (**Supplementary Figures 4**, **5**). The predicted scores for these epitopes ranged from 0.612 to 0.939 for NiV_1 (**Table 7**) and from 0.507 to 0.88 for NiV_2 (**Table 8**).

**Table 7:** List of the predicted conformational epitopes of NiV_1 by ElliPro tool of IEDB.

Residues	Number of residues	Score
R461, P462, T463, G464, P465, G466, P467, G468, K469, R470	10	0.939
M334, R337, G338, D339, K340, A341, Q342, E343, E344, M345, E346, G347, K348, Y350, T351, S352, D353, D354, E355, E356, A357, D358, Q359, L360, E361, F362, E363, D364, E365, K366, K367, V368, S369, Y370, S371, L372, K373, K375, E376	39	0.879
C30, N35, T36, Q37, I38, Y39, T40, L41, N42, D43, K44, I45, F46, S47, Y48, T49, E50, S51, L52, A53, G54, K55, R56, E57, M58, A59, I60, I61, T62, F63, K64, N65, G66, A67, I68, F69, Q70, V71, E72, V73, P74, G75, S76, Q77, H78, I79, D80, S81, Q82, K83, K84, A85, I86, E87, R88, M89, K90, D91, T92, L93, R94, I95, A96, Y97, L98, T99, E100, A101, K102, V103, E104, K105, L106, C107, V108, W109, N110, N111, K112, T113, P114, H115, A116, I117, A118, A119, I120, S121, M122, A123, N124, E125, A126, A127, A128, K129, A130, S131, F132, S133, W134, D135, T136, M137, I138, K139, A140, A141, Y142, Q143, T144, A145, E146, N147, P148	115	0.754
V418, L420, D421, S422, N423, G424, P425, G426, G428, F429, L500, E501, F502, R503, G504, P505, G506, P507, G508, A509, S510, F511, R512, S513, Y514, Q515, S516, K517, L518, G519, R520, D521, G522, R523, G524, P525, G526, P527, E552, D553, E554, F555, A556, G557, S558, L577, I578, N579, L580, D581, M582, R583, G584, P585, G586, P587, G588, K589, S590, D591, I592, K593, Y594, Q595, P596, L597, I598, S599, R600, I614, A615, T617, P618, G619, F620, P621, I622, S623	78	0.706
G198, V199, I200, S201, K202, A203, A204, Y205, Q206, Q207, K208, R209, V210, N211, T255, E256, F257, N258, P259, H260	20	0.676
G404, P405, G406, P407, G408, D409, R410, I411, N412, I414, S415, A416, I433, I434, V435, E436, K437, K438, R439, N440, T441, Y442, S443, G444, P445, G446, N450, T451, Y452, S453, E456, R459	32	0.626
K472, I473, R474	3	0.612

**Table 8:** List of the conformational epitopes of NiV_2 by ElliPro tool of IEDB.

Residues	Number of residues	Score
G1, I2, I3, N4, T5, L6, Q7, K8, Y9, Y10, C11, R12, V13, R14, G15, G16, R17, C18, A19, V20, L21, S22, C23, L24, P25, K26, E27, E28, Q29, I30, G31, K32, C33, S34, T35, R36, G37, R38, K39, C40, C41, R42, R43, K44, K45, E46, A47, A48, A49, K50, Q51, T52, A53, E54, N55, P56, V57, F58, T59, V60, A61, A62, Y63, A64, S65, F66, S67, W68, D69, T70, M71, I72, K73, A74, A75, Y76, S77, I78, V79, P80, N81, F82, I83, L84, V85, A86, A87, Y88, A89, Q90, I91, T92	92	0.883
F183, A184, A185, H187, T188, E189, F190, N191, P192, H193, N194, H195, Y196, K197, K198, I199, K200, S201, Y202, Y203, G204, T205, M206, D207, I208, K209, K210, I211, N212, E213, G214, K215, K216, F217, G218, L221, Q222, D223, P224, V225, S226, N227, S228, M229, T230, Q280, E284, K287, K288, Y291, S292, K294, E295	53	0.693
E422, R424, P426, G427, P428, G429, G447, P448, G449, L452, K455, A456, T458, A459, P460, D461, T462, A463, E464, G465, P466, G467, P468, G469, L470, E471, F472, E473, D474, E475, F476, A477, G478, S479, S480, S481, E482, V483, I484, G485, P486, G487, P488, G489, M490, G491, V492, I493, N494, S495, I496, K497, L498, I499, N500, L501, D502, M503, R504, G505, P506, G507, P508, G509, K510, S511, D512, I513, K514, Y515, Q516, P517, L518, I519, S520, R521, S522, N523, A524, G525, P526, G527, P528, G529, L530, L531, V532, S533, K534, I535, A536, Y537, T538, P539, G540, F541, P542, S544	98	0.678
T298, K299, A301, G302, R303, L304, G305	7	0.59
H166, E169, Q170, A173, Y174, I176, A177, P180	8	0.577
N156, F158, F159	3	0.553
K399, N401, T402, Y403, S404, G405, P406, G407, S411	9	0.533
G440, D442, G443, R444, G445	5	0.524
G365, P366, G367, P368, G369	5	0.507

### 3.10 Molecular docking analysis with TLR3

**Figure 5:**
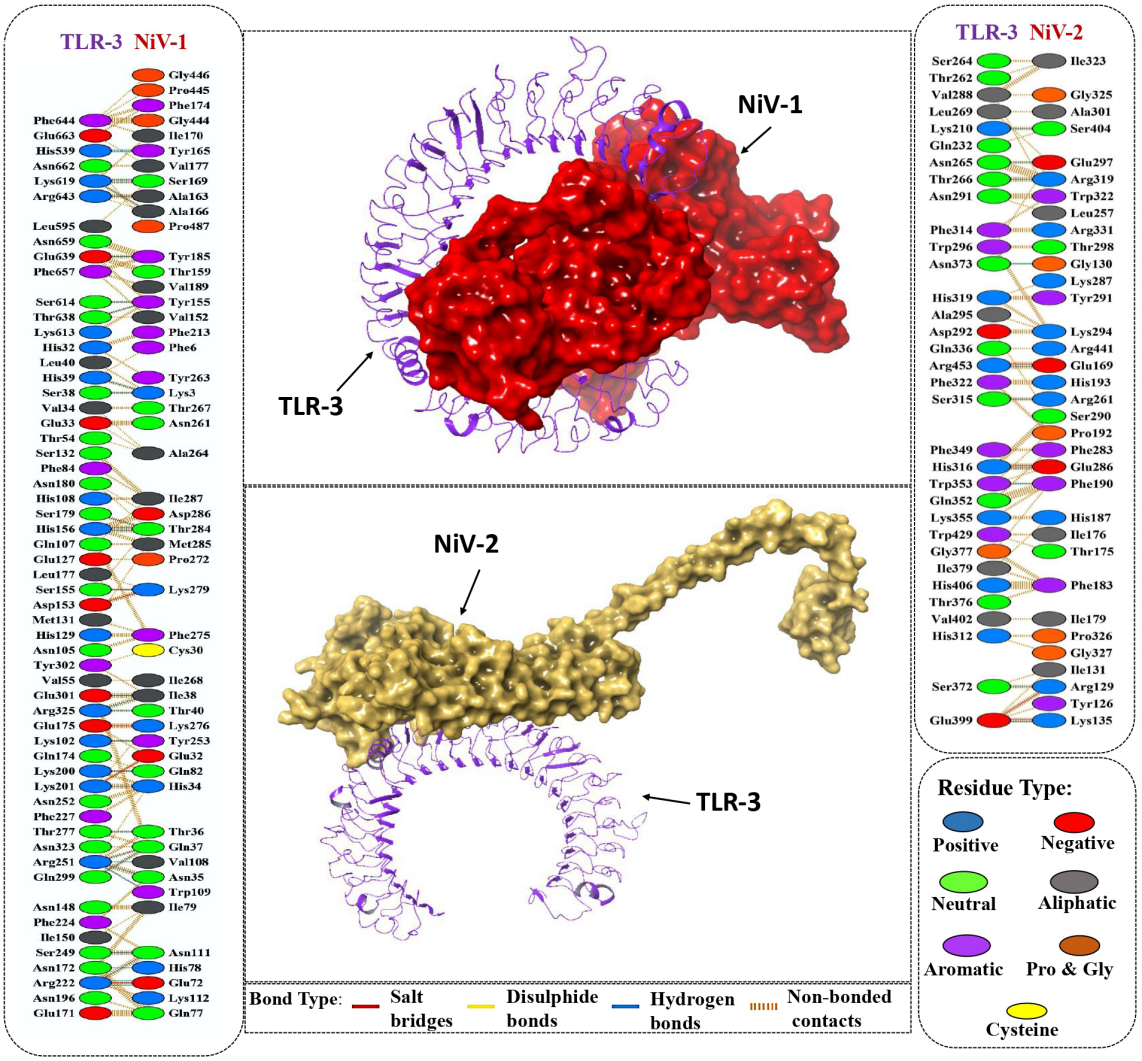
Molecular interaction of vaccine designs (NiV_1 & NiV_2) with human TLR-3. Cartoon depiction of and TLR-3 (Purple) and the surface presentation of vaccine construct NiV-1 (Red) and NiV-2 (Yellow). Detailed Atomic interactions at the interface between the vaccine constructs and TLR-3.

TLR3 is a member of the toll-like receptor family that recognizes double-stranded RNA and activates downstream signaling to induce antiviral immune responses (76). The Nipah virus has evolved a process to block the TLR3 signaling pathway, leading to a compromise in the host’s ability to defend against viruses (77). To overcome this evasion strategy, it is crucial to assess the ability of the designed vaccine to bind to the TLR-3 immune receptor via molecular docking analysis using ClusPro 2.0 webserver. Docking simulations generated 10 potential complexes for each vaccine construct, with varying energy scores. The final complexes for NiV_1 and NiV_2 were selected based on the lowest energy scores of -1284.8 kcal/mol and -1222.6 kcal/mol, respectively, indicating strong binding affinity between the designed vaccines and immune receptors. The molecular interactions between the proposed vaccines and the TLR receptor are depicted in **Figure 5**. The NiV_1 complex exhibited robust interactions, including 4 salt bridges, 32 hydrogen bonds, and 343 non-covalent interactions. Similarly, the NiV_2 vaccine formed 4 salt bridges, 10 hydrogen bonds, and 182 non-covalent interactions with the TLR3 receptor. The detailed atom-level interactions between TLR3 and NiV_1 & 2 are provided in **Supplementary Tables 1**, **2**.

### 3.11 Molecular dynamics simulation analysis of vaccine -TLR3 docked complex

**Figure 6:**
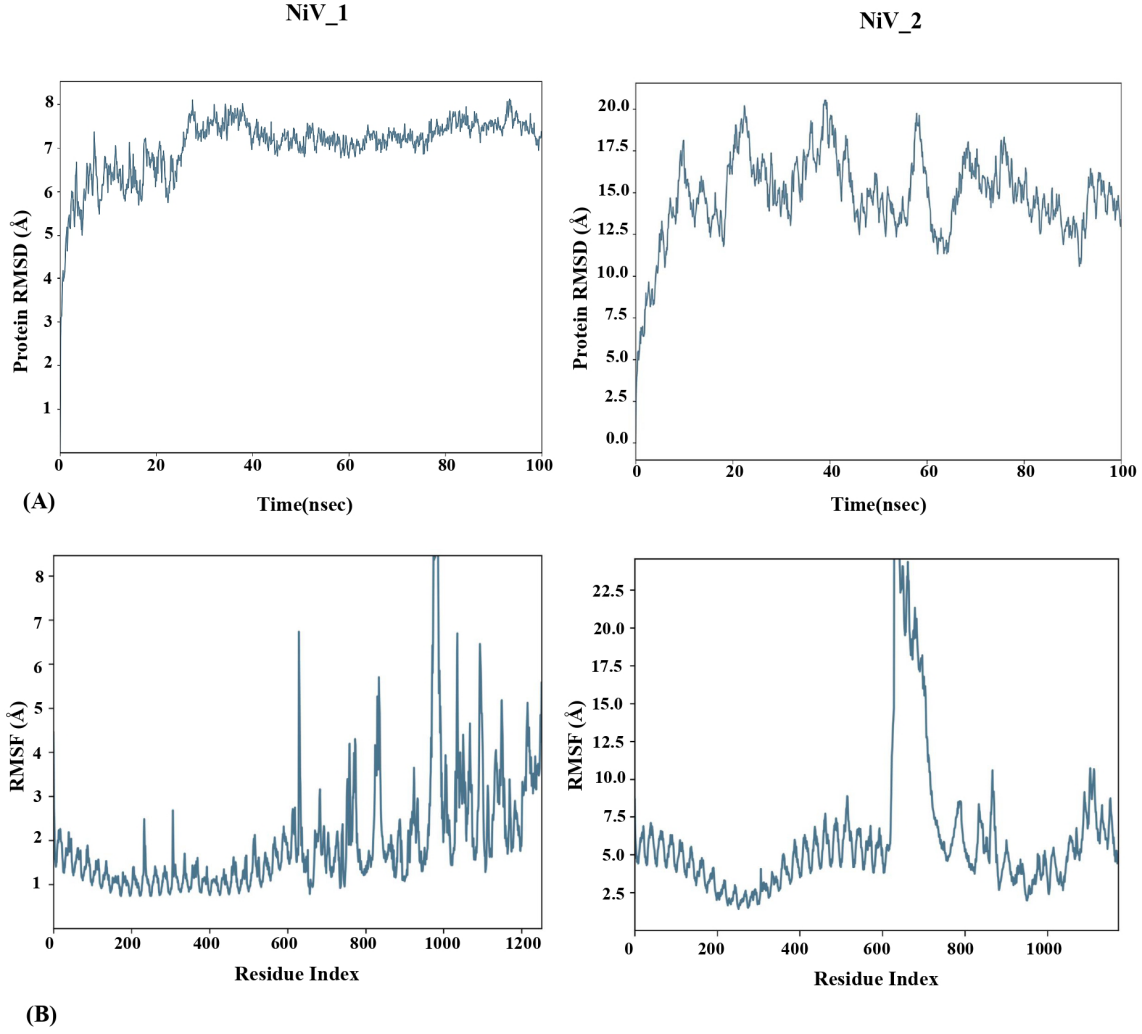
MD simulation study of TLR-3-Vaccine complexes for 100ns using Desmond Schrodinger Software. RMSD **(A)** and RMSF **(B)** analysis of TLR3-vaccine docked complex.

To determine the structural stability and the dynamic behavior of the TLR3-vaccine docked complexes, molecular dynamic simulation was performed for 100ns using Desmond Schrodinger software. For TLR3-NiV_1 docked complex, Root mean square deviation (RMSD) analysis showed variation up to about 25 ns; thereafter the docked vaccine-TLR3 complex maintained stability with less than 1Å deviation. In contrast, the TLR3-NiV_2 complex exhibited fluctuations (RMSD ≥ 9Å) throughout the simulation, implying its instability compared to the TLR3-NiV_1 complex (**Figure 6A**). These findings align with the docking analysis, indicating stronger binding interactions between NiV_1 and TLR3. Additionally, Root-mean-square fluctuations (RMSF) of the complexes was also performed to identify the flexibility across the amino acid residues in the TLR3-vaccine complexes. A high degree of fluctuations were observed in both of the vaccine molecules compared to the TLR3 molecule (**Figure 6B**), which exhibited mostly rigidity and low RMSF values. The average RMSF of TLR3-NiV_1 complex was calculated as 2.62Å, with ASP354 (13.02 Å; vaccine residue) showing highest fluctuation among residues analyzed (**Figure 6B**). The TLR3-NiV_2 complex demonstrated flexibility throughout the simulation, with RMSF values ranging 2 to 6Å (**Figure 6B**). Overall, the molecular dynamics simulation suggests that the NiV_1 multiepitope vaccine construct demonstrates stronger and more stable interactions with the TLR3 immune receptor. The study also indicates that residues in the adjuvants, CTL, B-cell, and HTL epitopes show increased flexibility, which seems to be essential for the designed vaccine to adopt a suitable conformation for interacting with immune cells.

### 3.12 In-silico cloning and codon optimization

**Figure 7:**
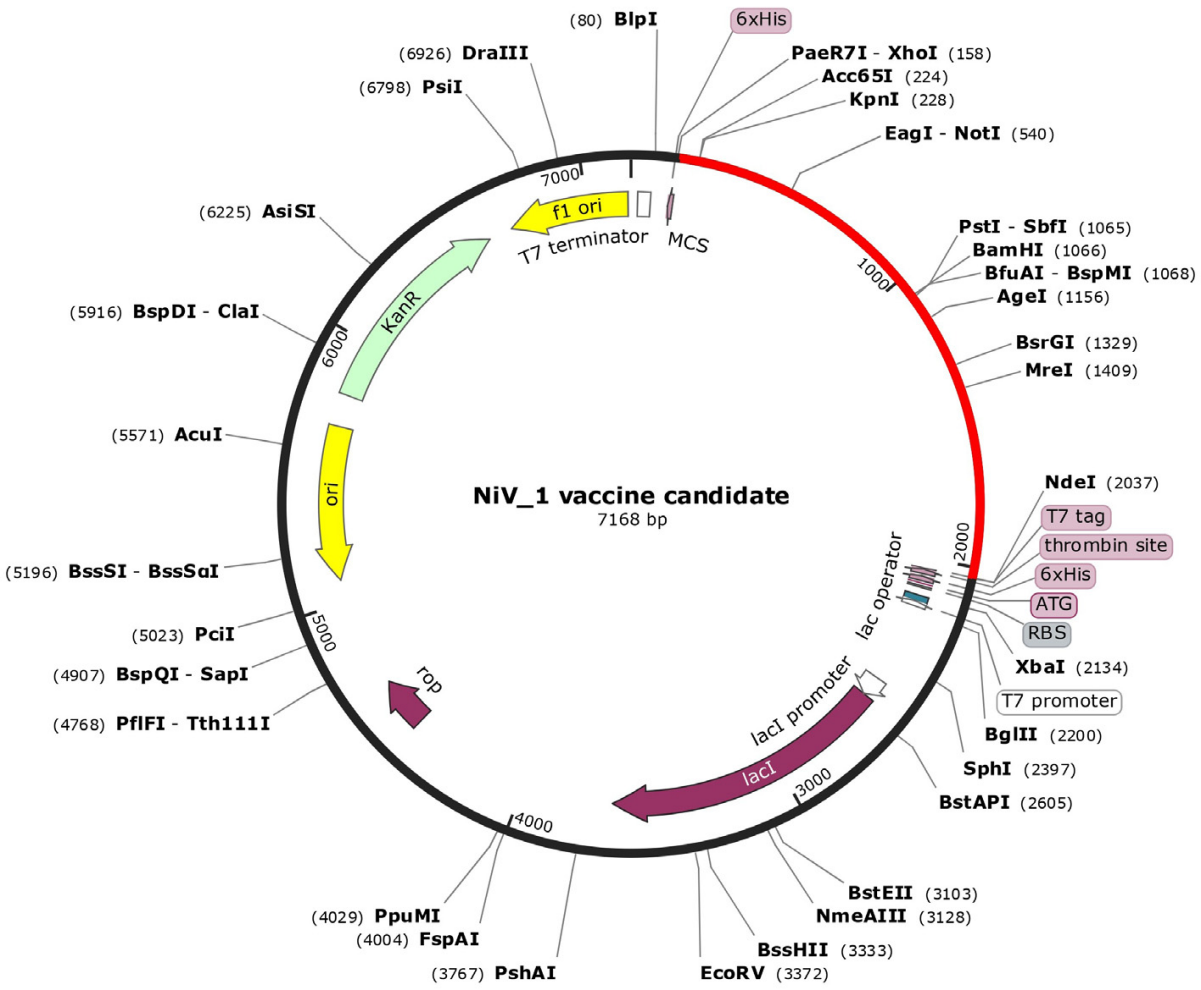
*In-silico* cloning of the designed vaccine construct in the pET28a + expression vector performed using SnapGene tool. The cloned construct (NiV_1) is represented by the red arc segment, while the remaining segment represents the vector backbone.

After analyzing both vaccine constructs, we finalized the NiV_1 vaccine construct based on the molecular docking and molecular dynamics simulation results. The designed vaccine construct’s reverse translation and codon optimization were conducted using the VectorBuilder Codon optimization tool. Utilizing the Codon optimization web tool, an assessment of significant gene sequence properties for achieving high-level protein expression in the E. coli host was performed, including estimation of Codon Adaptation Index (CAI) and GC content. The optimized codon sequence comprised 1872 nucleotides. A codon adaptation index value greater than 0.8 and a GC content ranging from 30% to 70% are considered conducive for effective protein expression in the host system. The analysis revealed that the designed vaccine construct obtained a CAI value of 0.94, with a desirable GC content of 53.85% that can help achieve high protein expression. Furthermore, restriction sites XhoI and NdeI were incorporated into both the N and C terminals of the final vaccine codon sequence before being inserted into the pET-28a(+) vector using the SnapGene tool (**Figure 7**). The graphical representation in **Figure 7** depicts the successful cloning of NiV_1 vaccine construct into the pET-28a(+) expression vector.

### 3.13 Immune system simulation analysis

**Figure 8:**
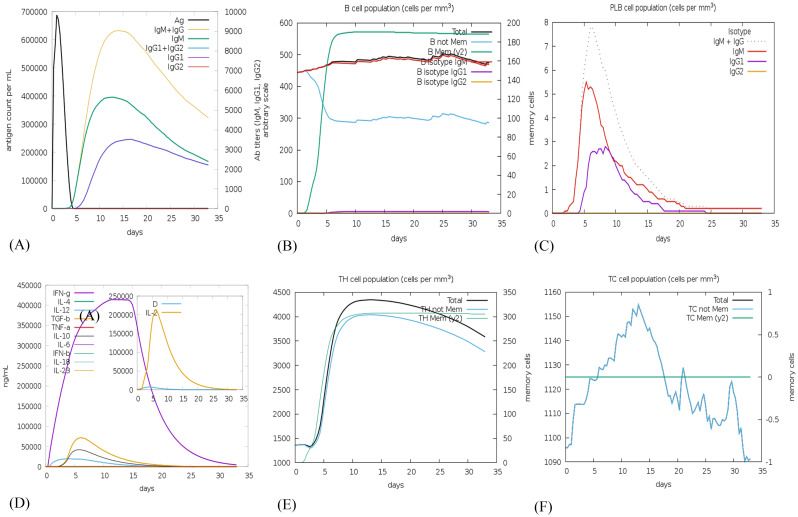
*In silico* immune simulation using the c-ImmSim server following a single vaccine injection of NiV_1 **(A)** Generation of immunoglobulins and formation of immune complexes, **(B)** B-cell population, **(C)** Count of plasma B lymphocytes by isotype (IgM, IgG1, and IgG2), **(D)** Cytokine production, **(E)** T-helper cell population levels (TH), and **(F)** Cytotoxic T-cell population levels.

The C-ImmSim web server was utilized to forecast the immune response profile for the suggested vaccine construct. The findings indicate that the construct has the potential to trigger an immune reaction. The graph demonstrates that within 5 days of reaching approximately 700,000 antigens per mL, the antigen is eliminated from the host system. It is evident that during the first 15 days following injection when antigen count was close to zero, there was a rise in production rate of primary antibodies as well as IgM and IgG levels increased up to around 9000. Subsequently, although the complex count reduced, it stabilized at approximately 4500 after one-month indicating sustained and long-lasting immunity response (**Figure 8A**). Following vaccination, the total B cell count exceeded 450 cells/mm³, and memory B cell and IgM levels persisted throughout the simulation (**Figure 8B**). Plasma B cell levels reached a peak of over 7 cells/mm³ between days 5 and 10, with subsequent elevations in IgM and IgG1 levels. Notably, **Figure 8C** highlights a substantial increase in plasma B cell production, underscoring its critical role in eliminating pathogens.

The vaccine construct was also examined for its ability to induce cytokine production including interferon gamma, interleukins etc. **Figure 8D**, depicts the release of 400000 and 200000 ng\mL concentration of interferon gamma and interleukins, respectively, generated by the injection of the proposed vaccine candidate. Additionally, the populations of helper T (Th) and cytotoxic T (Tc) cells significantly increased post-vaccination, as depicted in **Figures 8E, F**. According to C-ImmSim simulations, the potential NiV_1 vaccine construct can induce a durable cellular and humoral immune response. Nevertheless, experimental validation is imperative to confirm its ability to elicit adaptive immunity against NiV_1.

## 4 Discussion

Vaccination plays a crucial role in reducing the impact of new viruses on public health and worldwide stability. Recent occurrences of new diseases like the Ebola virus, Zika virus, SARS-CoV-2 and Nipah virus have highlighted the substantial risks that emerging viruses present to human health. Vaccines serve as indispensable tools in activating the host immune system and inhibiting the attack of various pathogen-borne infectious diseases, thereby providing protection to both individuals and communities. Despite being one of the most effective preventive measures against infectious agents, there is currently no approved vaccine for preventing NiV viral disease. Rapid advancements in immuno-informatics methodologies have emerged as crucial contributors in reducing costs and time, while simultaneously enhancing the precision of epitope-based vaccine development (35, 78). Several research studies have employed immunoinformatics methods to design vaccines for the various viral disease such as SARS CoV-2 (79–81), Influenza virus (82), Lassa virus (83), Herpes simplex virus (84, 85), Yellow Fever Virus (86), Chikungunya (87), and Dengue virus (88) etc. Over the past decades, a significant portion of studies has predominantly focused on single or dual proteins of NiV for the designing of multi-epitope vaccines (89–94). As widely acknowledged, the Nipah virus (NiV), being an RNA virus, demonstrates a propensity for undergoing spontaneous mutations. These mutations can potentially expedite the escape of the virus from immune selection, especially if the vaccine is designed to target only one or two antigens. Therefore, it is crucial to develop a multi-epitope vaccine that targets multiple antigens in order to ensure comprehensive protection against the Nipah virus and to minimize the probability of escape mutations.

The goal of the study is to formulate a multi-epitope vaccine that targets the multiple antigenic structural proteins of the NiV. This research marks the first instance of not only designing two distinct vaccine constructs using different adjuvants, but also executing a comparative analysis through a bioinformatics methodology. This approach leverages various in-silico and immunoinformatics techniques to enhance the likelihood of achieving a robust and durable immune response. In this current investigation, we initially obtained the protein sequences from NCBI and conducted an analysis focused on antigenicity. Following the antigenicity examination, these sequences were then subjected to epitope prediction in order to stimulate both B-cell and T-cell mediated immunity using a wide array of immunoinformatics approaches. Additionally, the identification of the potential vaccine candidate CTL involved the assessment of various parameters such as antigenicity, allergenicity, toxicity, conservancy, immunogenicity (for CTL epitopes), and IFN-gamma inducer (for HTL epitopes only). By selecting T-cell epitopes that have the ability to interact with multiple HLA-I/II alleles, a broader population coverage can be achieved, thereby increasing the chances of an effective immune response across diverse populations. Following the evaluation process, 12 CTL, 12 HTL, and 6 linear B-cell epitopes were chosen to design the vaccine construct, aiming to activate both primary and secondary immune responses. An analysis of T-lymphocyte epitopes and their distribution of MHC alleles worldwide indicated a global coverage of 99.99%. Additionally, the regions most affected by NiV exhibited a higher degree of coverage, as predicted by the population coverage analysis (**Figure 2**).

The epitopes that were examined were connected by various linkers, namely AAY, KK, and GPGPG. The utilization of linkers in the context of a multiple epitope vaccine offers a significant advantage by preventing the formation of junctional antigens and enhancing the processing and presentation of antigens (95). An EAAAK linker was utilized to provide structural rigidity, reducing hindrance during the interaction of the adjuvant and its receptor (96). The choice of these linkers was primarily based on their ability to serve as proteasomal cleavage sites (AAY), elicit a helper T-cell response (GPGPG), and maintain the immunogenicity of B cells (KK), while also adjusting the pH closer to the physiological range (96–98). The efficacy of a peptide-based vaccine significantly relies on the adjuvant used in the subunit formulation. Adjuvants have been previously reported as immunomodulatory agents capable of enhancing the efficacy of multiple vaccine constructs (99, 100). Moreover, immune responses to adjuvants do not necessarily compromise vaccine efficacy and may instead improve antigen-specific immunity. To determine a suitable adjuvant, an evaluation was conducted on two peptides: Beta-defensin and Cholera toxin. Extensive research supports the role of Beta-defensin and Cholera toxin as immune modulators against various pathogens (101–104). Beta-defensin is an antimicrobial peptide that plays a crucial role in innate immune mechanisms through TLR-3 activation (105). Beyond its antiviral activity, β-defensin also stimulates adaptive immunity by recruiting naive T-cells and immature dendritic cells (DCs) to infection sites (106). Additionally, The Cholera toxin B subunit is a potent mucosal adjuvant that enhances the uptake and presentation of antigens by diverse immune cells. Leveraging its strong binding affinity for the monosialotetrahexosyl ganglioside receptor, which is expressed on various cell types such as epithelial cells, antigen-presenting cells, macrophages, dendritic cells, and B-cells, CTB has been extensively employed in vaccine design to strengthen immune responses (107, 108). Both CTB and Beta-defensin 3, which have been experimentally validated as potential viral adjuvants (109–113), were incorporated into the construction of two different vaccine formulations to enhance the immune response. Overall, In comparison to conventional adjuvants, CTB and Beta-defensin 3 confer unique immunological benefits, rendering them particularly advantageous for mucosal-targeting vaccines. The designed vaccines were found to be antigenic, non-allergenic and non-toxic in nature. Furthermore, it is crucial to evaluate the physiochemical characteristics of the engineered vaccine construct in order to determine its safety and efficacy as a multi epitope vaccine (114). Evaluation of these properties was performed using ExPASy ProtParam, which revealed a molecular weight of 68 kDa for NiV_1 and 59kDa for NiV_2, respectively. It is worth noting that proteins with a molecular weight below 110 kDa are known to exhibit faster expression and simpler purification compared to their heavier counterparts (113). Additionally, the analysis indicated stability, hydrophilicity, and thermostability, thereby resulting in a longer half-life in both *in vitro* and *in vivo* experimental settings. The construct also demonstrated appropriate solubility upon expression (refer to **Table 5**).

Analysis of the secondary structure revealed the proportions of alpha helix, beta-sheet and coil. Coils were the predominant structure in the vaccine constructs. The tertiary structure of the proposed vaccine candidates were predicted and further refined using the RoseTTAfold and GalaxyRefine, respectively. Validation of the 3D refined structures were performed using PROCHECK, ERRAT and ProSA-web server. The best model that was selected for structural analysis, confirmed the stability of the vaccine constructs and demonstrated the high proportion of residues in the favorable region of the Ramachandran plot. Additionally, the analysis demonstrated that the ERRAT and Z-scores for NiV_1 and 2 constructs lie within the expected range of scores (X-ray, NMR) seen in comparable-sized native proteins (60, 64), providing further validation of the reliable structural integrity. Notably, the ElliPro tool predicted 7 and 9 conformational epitopes on the NiV_1 and NiV_2 vaccine structures, respectively.

The vaccine must possess the capability to attach itself to innate immune receptors in order to activate innate immunity and hinder tolerance (115). Diverse TLRs play a role in the initial interaction between host cells and invading viruses, controlling both virus replication and host responses, ultimately impacting the virus’s pathogenesis (116). TLR-3, also known as toll-like receptor 3, plays a crucial role in the activation of the immune response against the virus, signal transduction, and the induction of IFN release (117). In this study, we utilized molecular docking analysis and MD simulations to evaluate the effectiveness and stability of the designed vaccines with TLR3 immune receptor. The underlying concept behind this analysis is to assess the interaction between the vaccination and the target immune cells, which ultimately leads to the development of a strong immunological response in the host. The docking analysis indicated that the TLR3-NiV_1 complex displayed the most favorable binding affinity, as evidenced by its lowest binding free energy. Furthermore, MD simulations confirmed the superior stability and flexibility of the TLR3-NiV_1 complex compared to the TLR3-NiV_2 complex.

In order to achieve optimal production of a recombinant vaccine protein in E. coli (strain K12), we conducted codon optimization to enhance both transcriptional and translational efficiency (118). To enhance protein expression in E. coli K12, the protein sequence of the vaccine constructs underwent codon optimization and was subsequently reverse translated into its specific cDNA sequence. The vaccine’s GC-Content of 53.85% suggests a high probability of successful expression in E. coli. While high expression levels are beneficial, there is a risk of misfolding or aggregation, which can compromise vaccine efficacy (119). Therefore, balancing expression optimization with structural integrity is critical for successful vaccine development. For convenient in silico cloning, the vaccine was inserted into the expression vector pET-28a (+), allowing its expression in a bacterial system (**Figure 7**).

For the immune simulation, the C-ImmSim tool was employed to simulate the in-silico immune response of the human body, allowing for a comprehensive understanding of the actual response generated by our immune system. After the administration of a single injection, there was a remarkable elevation in the levels of Ig production, IFN-γ, and IL-2, B and T-cell production underscoring the potential of the vaccine candidates to stimulate the immune system (**Figure 8**). Our finding highlights the potential of designed vaccine construct to induce a robust immune response and provide protection against NiV.

## 5 Conclusion

The recurring Nipah virus outbreaks and the lack of effective antiviral therapies, underscore the necessity for a safe and efficient multi-epitope vaccine. This investigation employed reverse vaccinology, immunoinformatics, and bioinformatics approaches to design vaccine candidates targeting NiV structural proteins. The two designed vaccine constructs in this study, exhibited robust antigenicity, immunogenicity, non-allergenicity, and non-toxicity, while meeting key physicochemical criteria. Molecular dynamics simulations and molecular docking studies validated the stability of the NiV_1 vaccine construct (incorporating the CTB adjuvant) and confirmed its strong interactions with the TLR-3 receptor. Furthermore, the conservation of selected epitopes across NiV strains suggests the potential for cross-protection. Multiple analyses indicate that the developed vaccine model NiV_1 can effectively trigger both innate and adaptive immune responses against the targeted pathogen. While computational models provide valuable in-silico insights by simulating immune dynamics and predicting responses, they have inherent limitations that require experimental validation. Although these approaches offer a strong preliminary framework, empirical studies are essential to confirm the efficacy, specificity, and safety of the vaccine constructs.

## Data Availability

The datasets presented in this study can be found in online repositories. The names of the repository/repositories and accession number(s) can be found in the article/**Supplementary Material**.
