# Supplementary material for: Comprehensive immunoinformatics and bioinformatics strategies for designing a multi-epitope based vaccine targeting structural proteins of Nipah virus
Source: Front Immunol. 2025 May 13;16:1535322. doi: 10.3389/fimmu.2025.1535322 (PMC12106399; doi:10.3389/fimmu.2025.1535322)

Supplementary Material

**A Comprehensive Immunoinformatics and Bioinformatics Strategies for Designing a Multi-epitope Based Vaccine targeting structural Proteins of Nipah Virus.**

Shivangi Sharma^1^, Pragya D Yadav^1^, Sarah Cherian^1*^

^1^ICMR-National Institute of Virology, Pune, Maharashtra, India 411001.

*Correspondence: [sarahcherian100@gmail.com](mailto:sarahcherian100@gmail.com)

# Supplementary information

**Supplementary Figure 1. Population Coverage analysis.** The worldwide population coverage of combined CD8+ and CD4+ epitopes for the vaccine construct.

**Supplementary Figure 2.** **Secondary structure analysis of designed vaccine using Psipred server.** Graphical depiction of the secondary structure of the selected vaccine candidates NiV_1 (a) and NiV_2 (b), illustrating helices, coils, and strands.

**Supplementary Figure 3. Structural analysis of modeled 3D structure of NiV_1 & 2 vaccine constructs before refinement.** (a) Assessment of the vaccine construct's 3D structure through Ramachandran plot examination. (b) Evaluation of the anticipated model using ProSA-web (c) The ERRAT score of modeled structures of NiV_1 & 2.

**Supplementary Figure 4. Predicted conformational epitopes of NiV-1 vaccine construct.** Number of residues and score of are shown along with the 3D representation of each epitopes.

**Supplementary Figure 5. Predicted conformational epitopes of NiV-2 vaccine construct.** Number of residues and score of are shown along with the 3D representation of each epitopes.

# Supplementary Figures and Tables


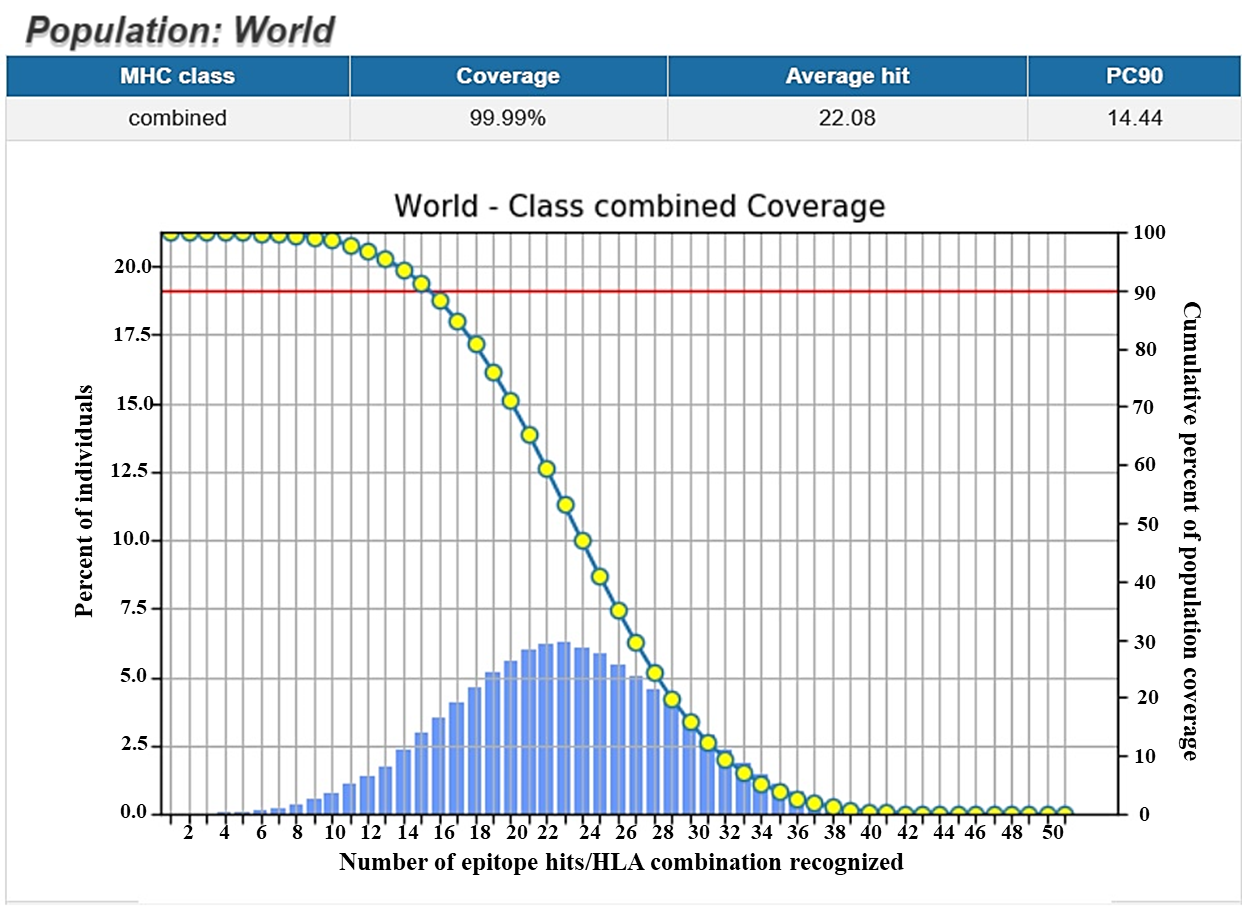


**Supplementary Figure 1.** Population Coverage analysis. The worldwide population coverage of combined CD8+ and CD4+ epitopes for the vaccine construct.

**
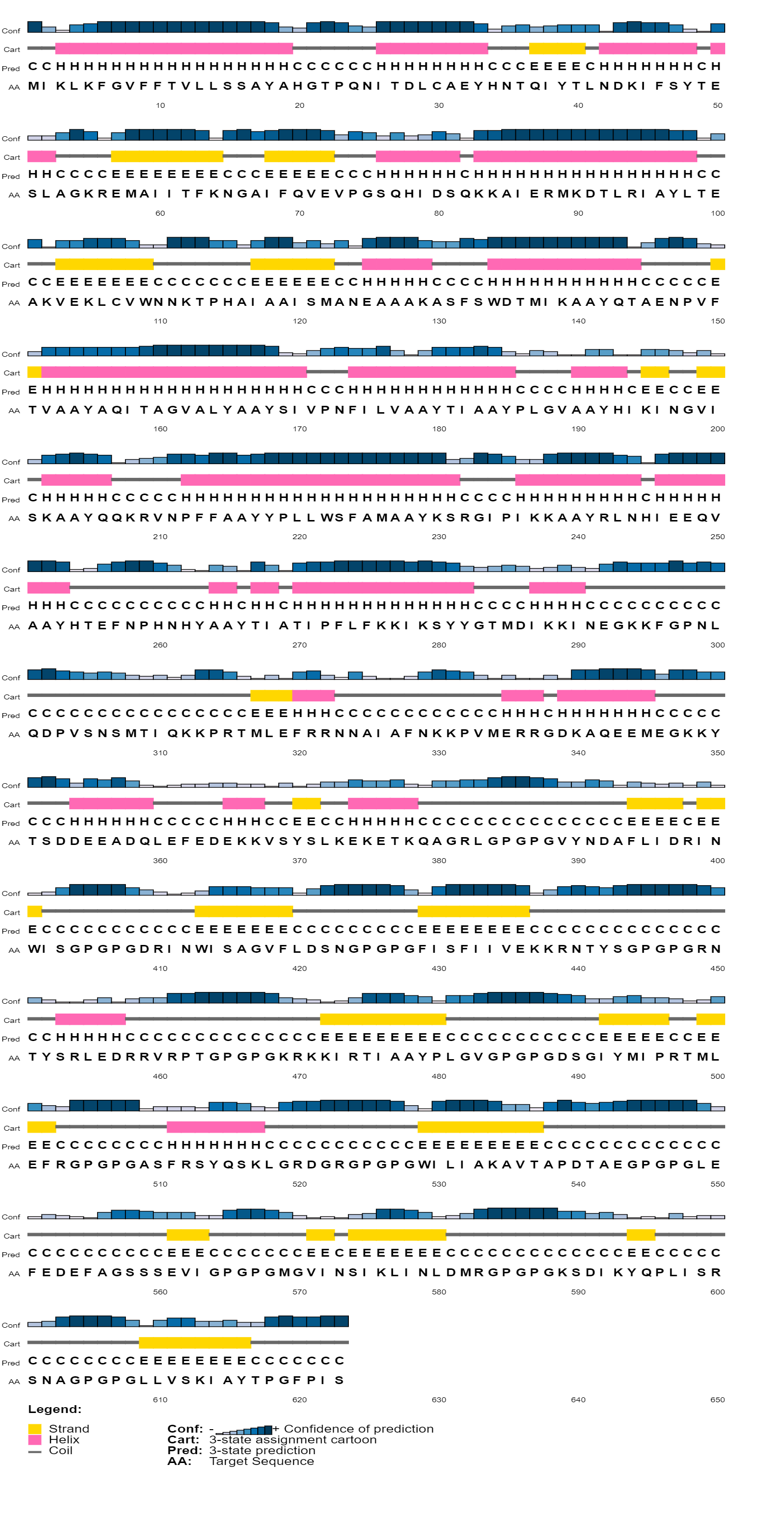
 Top of Form(A)**


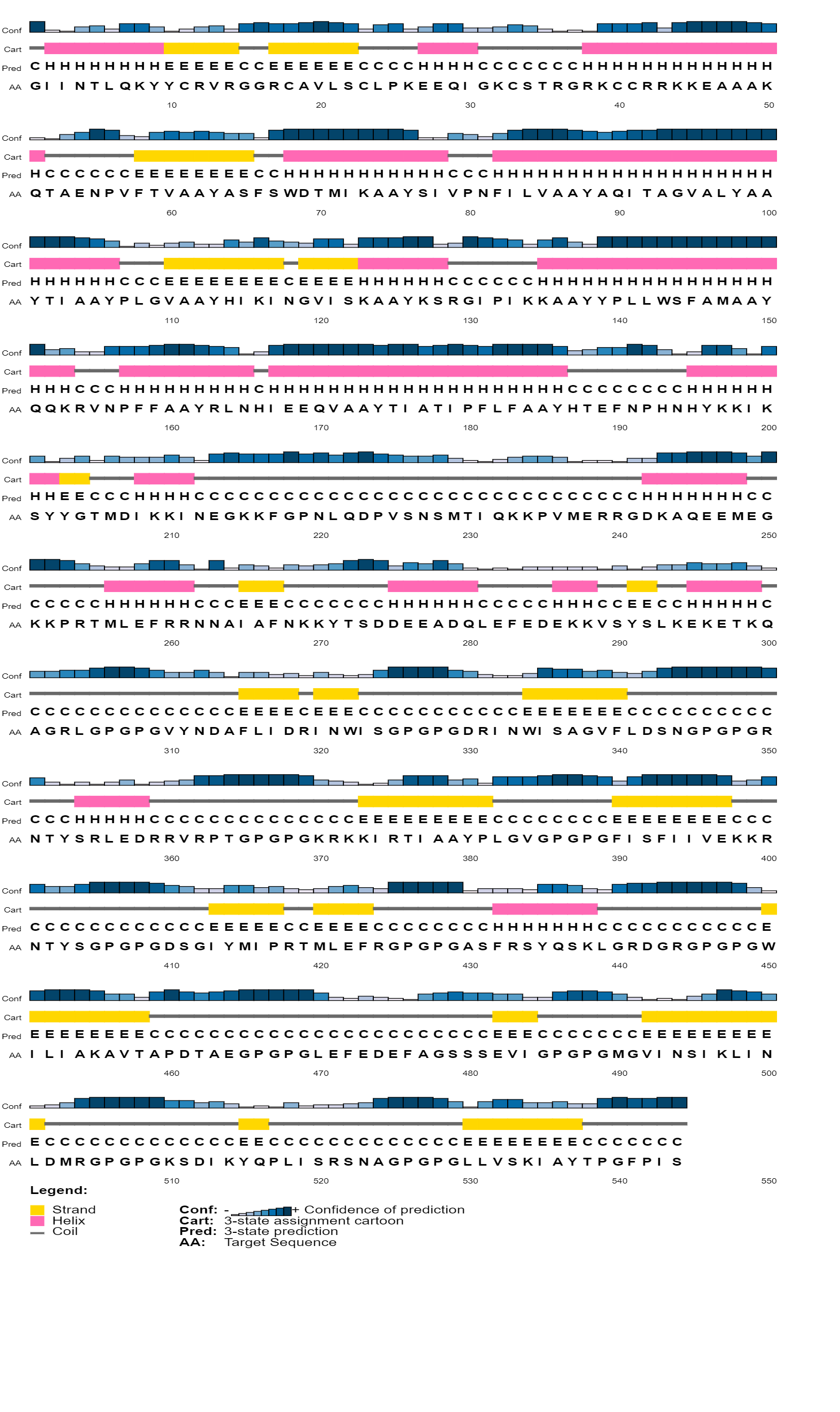

**(B)**

**Supplementary Figure 2.** Secondary structure analysis of designed vaccine using Psipred server. Graphical depiction of the secondary structure of the selected vaccine candidates NiV_1 (A) and NiV_2 (B), illustrating helices, coils, and strands.


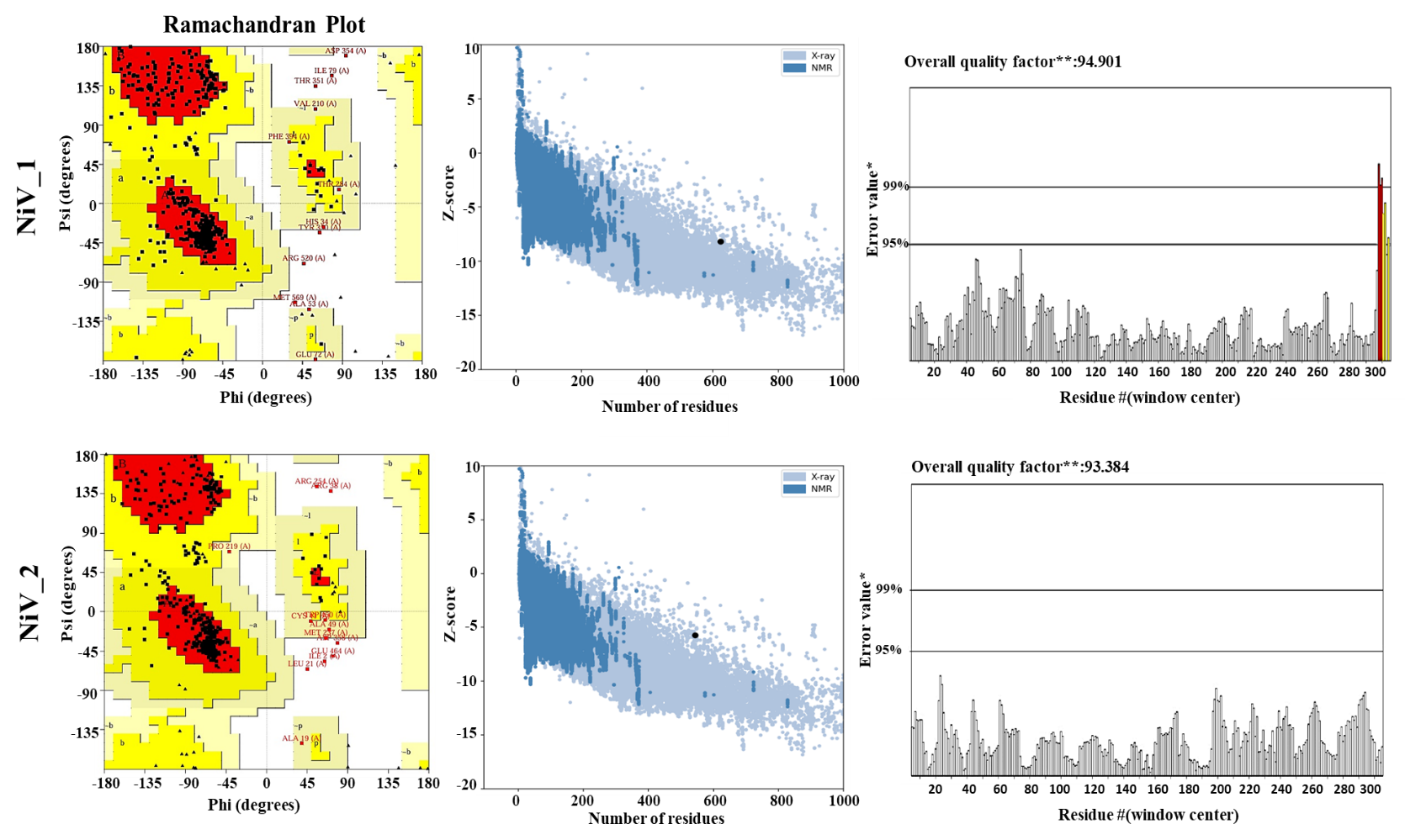


**Supplementary Figure 3.** Structural analysis of modeled 3D structure of NiV_1 & 2 vaccine constructs before refinement. (A) Assessment of the vaccine construct's 3D structure through Ramachandran plot examination. (B) Evaluation of the anticipated model using ProSA-web (C) The ERRAT score of modeled structures of NiV_1 & 2.

**
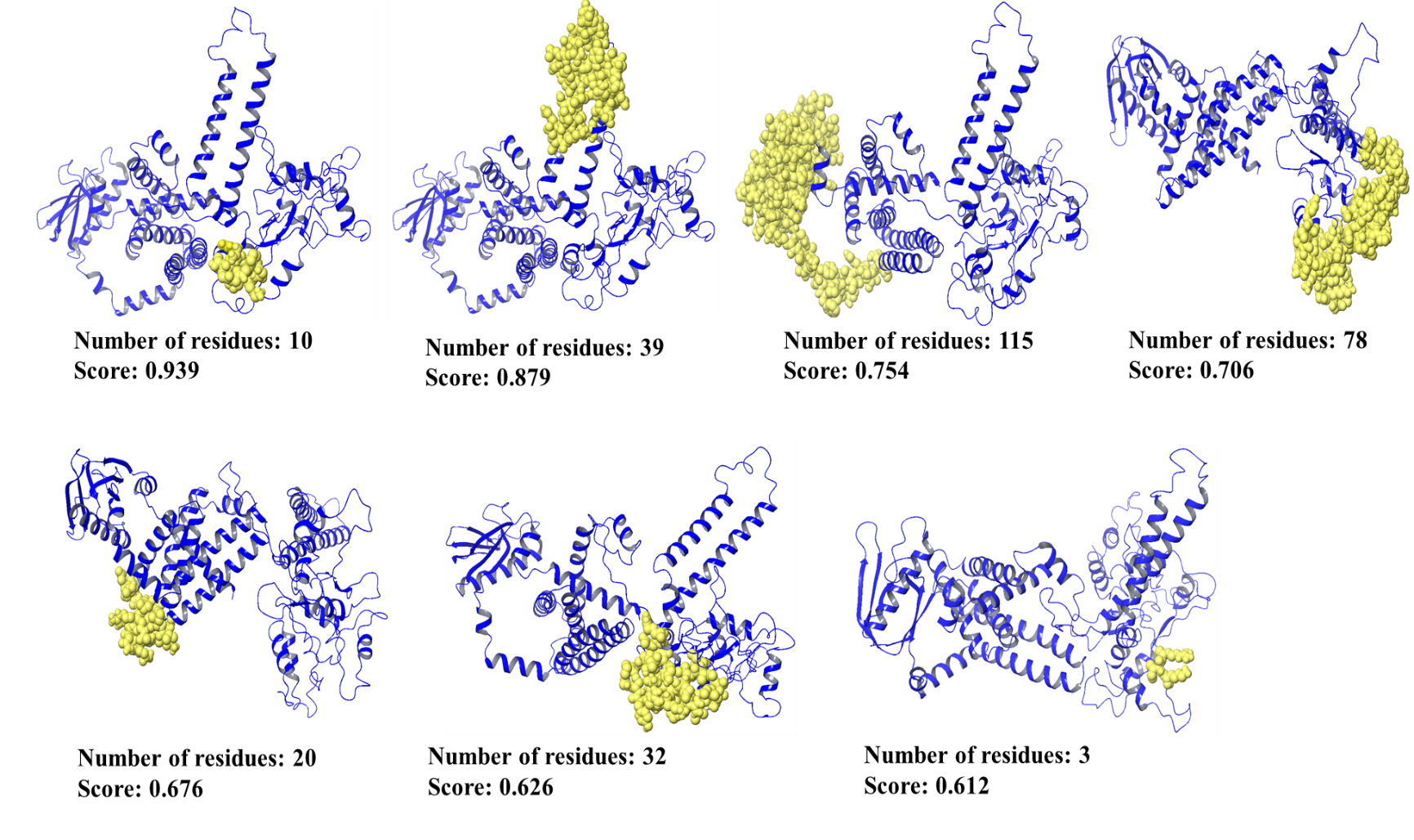
**


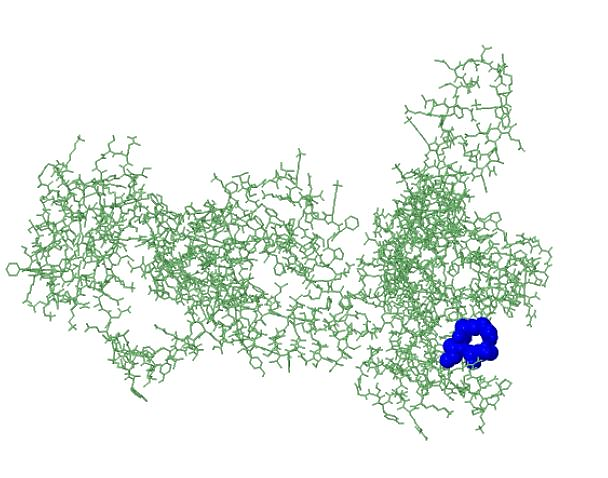

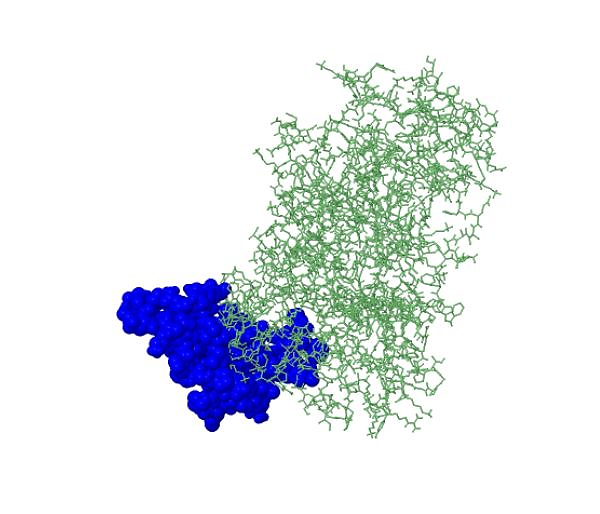

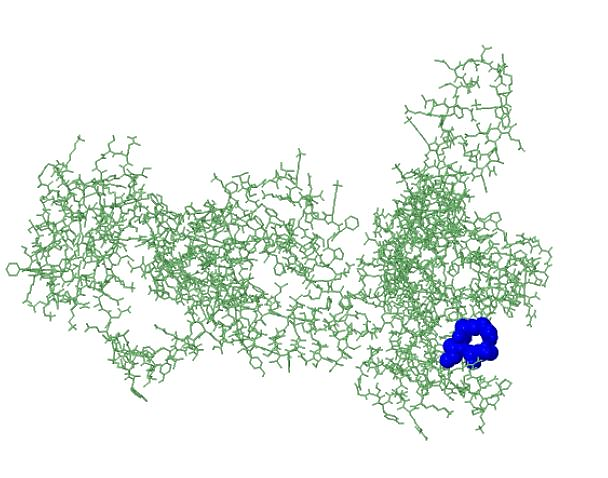

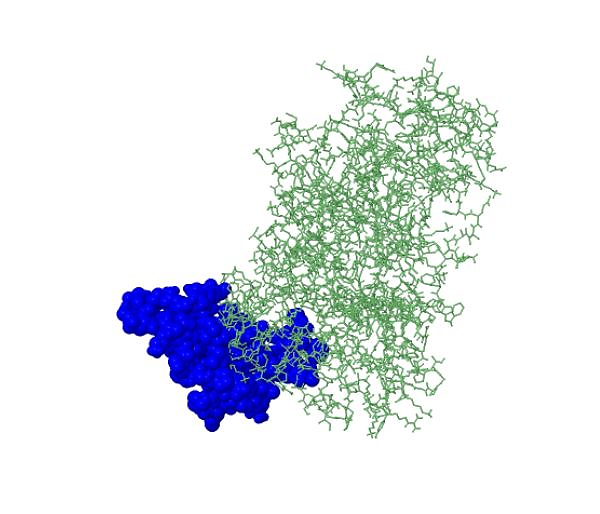
**
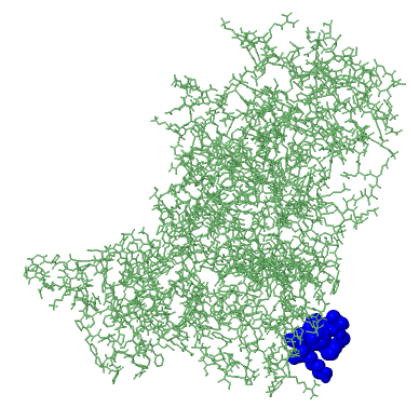

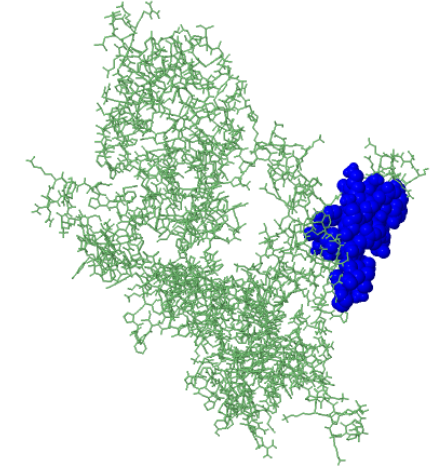

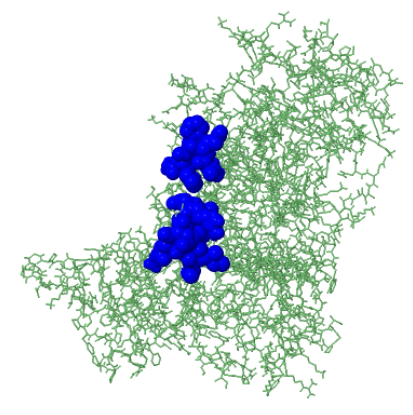

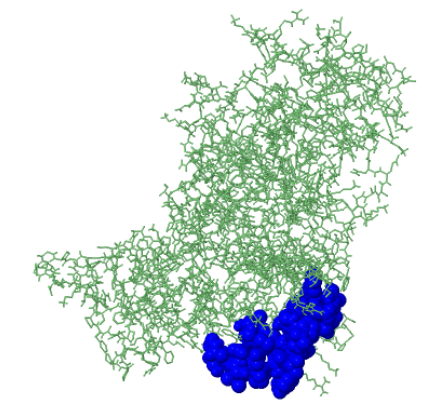
Supplementary Figure 4.** Predicted conformational epitopes of NiV-1 vaccine construct. The number of residues and corresponding scores are displayed, with each conformational B-cell epitope represented on the yellow surface.

**Supplementary Figure 5.** Predicted conformational epitopes of NiV-2 vaccine construct. The number of residues and corresponding scores are displayed, with each conformational B-cell epitope represented on the green surface.
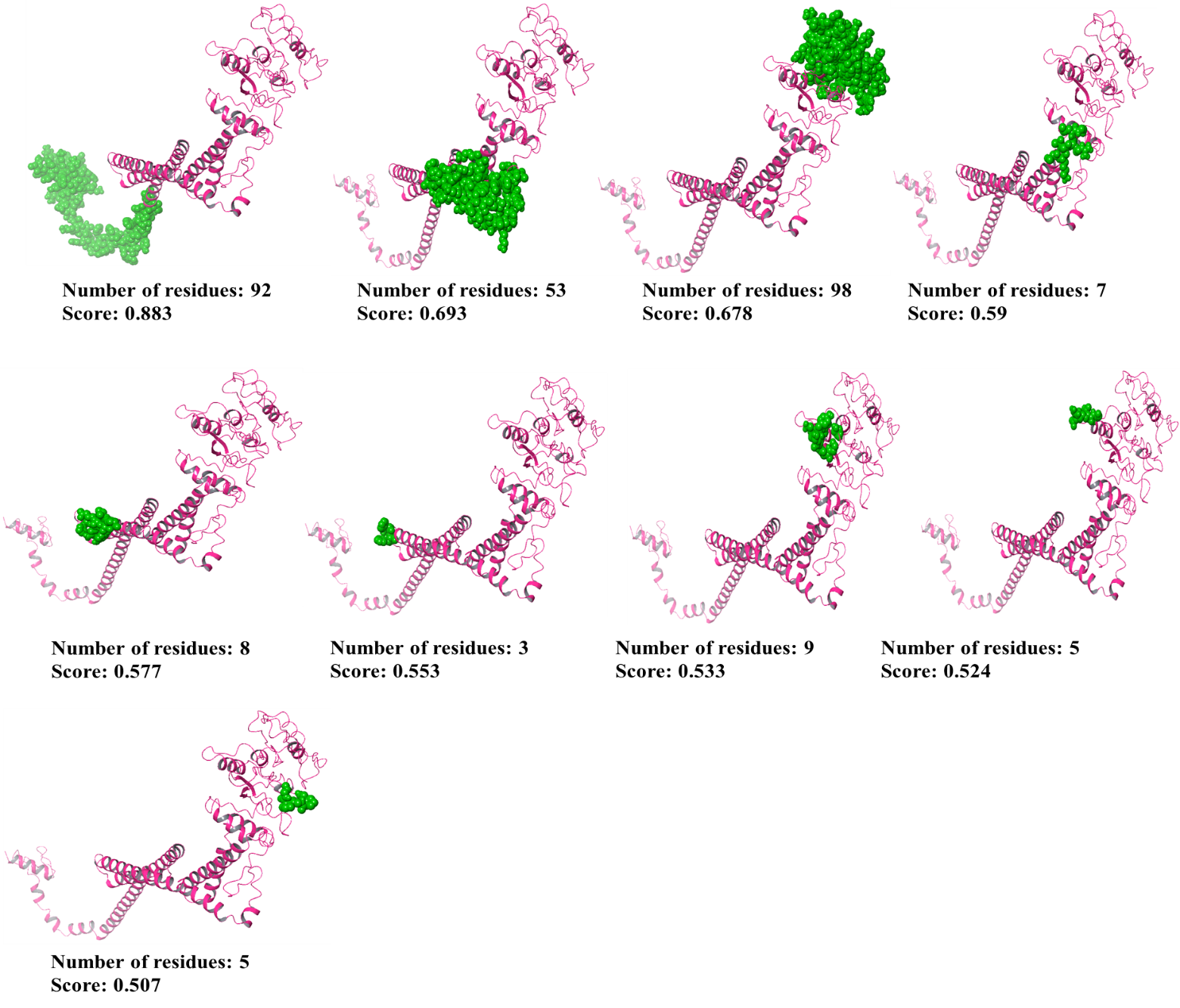

Supplement: Supplementary file 1 [file DataSheet1.docx]
